# Menin Deficiency Induces Autism‐Like Behaviors by Regulating *Foxg1* Transcription and Participates in *Foxg1*‐Related Encephalopathy

**DOI:** 10.1002/advs.202307953

**Published:** 2024-04-06

**Authors:** Kai Zhuang, Lige Leng, Xiao Su, Shuzhong Wang, Yuemin Su, Yanbing Chen, Ziqi Yuan, Liu Zi, Jieyin Li, Wenting Xie, Sihan Yan, Yujun Xia, Han Wang, Huifang Li, Zhenyi Chen, Tifei Yuan, Jie Zhang

**Affiliations:** ^1^ Institute of Neuroscience College of Medicine Xiamen University Xiamen Fujian 361105 China; ^2^ Department of Anesthesiology First Affiliated Hospital College of Medicine Xiamen University Xiamen Fujian 361105 China; ^3^ Shanghai Mental Health Center Shanghai Jiaotong University School of Medicine Shanghai 200030 China; ^4^ The Key Laboratory of Neural and Vascular Biology Ministry of Education College of Basic Medicine Hebei Medical University Shijiazhuang 050017 China

**Keywords:** ASD‐like behaviors, Atrx, FOXG1 syndrome, menin

## Abstract

*FOXG1* syndrome is a developmental encephalopathy caused by *FOXG1* (Forkhead box G1) mutations, resulting in high phenotypic variability. However, the upstream transcriptional regulation of *Foxg1* expression remains unclear. This report demonstrates that both deficiency and overexpression of *Men1* (protein: menin, a pathogenic gene of *MEN1* syndrome known as multiple endocrine neoplasia type 1) lead to autism‐like behaviors, such as social defects, increased repetitive behaviors, and cognitive impairments. Multifaceted transcriptome analyses revealed that *Foxg1* signaling is predominantly altered in *Men1* deficiency mice, through its regulation of the Alpha Thalassemia/Mental Retardation Syndrome X‐Linked (Atrx) factor. Atrx recruits menin to bind to the transcriptional start region of *Foxg1* and mediates the regulation of *Foxg1* expression by H3K4me3 (Trimethylation of histone H3 lysine 4) modification. The deficits observed in menin deficient mice are rescued by the over‐expression of *Foxg1*, leading to normalized spine growth and restoration of hippocampal synaptic plasticity. These findings suggest that menin may have a putative role in the maintenance of *Foxg1* expression, highlighting menin signaling as a potential therapeutic target for *Foxg1*‐related encephalopathy.

## Graphical abstract

*FOXG1* syndrome is a developmental encephalopathy resulting from the dysregulation of *FOXG1*. Here, it is reported that both deficiency and overexpression of *Men1* result in autism‐like behaviors by transcriptional regulation of *Foxg1* which is mediated by Atrx‐mediated H3K4me3 modification. This work reveals a putative role of menin in maintaining *Foxg1* expression which will benefit the *Foxg1*‐related encephalopathy therapeutic intervention development.

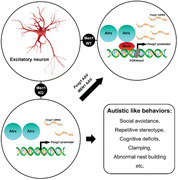

## 1 Introduction

*FOXG1* syndrome is a major developmental encephalopathy that belongs to the autism spectrum disorder (ASD). It exhibits both classical and atypical ASD phenotypes, including severe microcephaly (postnatal or congenital) and neurodevelopmental delay.^[^ ^1^ ^]^ FOXG1 is a crucial member of the Forkhead gene family and plays a vital role in brain development. *FOXG1* haploinsufficiency in humans is associated with significant differences in brain size and impaired intellectual development in early childhood, while homozygous mutations are typically fatal.^[^ ^2^ ^]^ Despite the crucial roles of *FOXG1* in the nervous system, limited knowledge exists regarding the upstream regulation mechanisms governing the *FOXG1* gene. Interestingly, patients with micro‐fragment deletion of 11q13.1, where the *MEN1* gene is located, exhibit autism‐like behaviors.^[^ ^3^ ^]^ This clinical observation implies a strong association between menin and neurodevelopmental disorders. We previously found that neuronal specific deletion of *Men1* leads to neuronal impairments in dendritic branching, spine density and synaptic plasticity, as well as ASD‐like behaviors in mice (*Men1*‐CcKO mouse). To investigate the molecular mechanism mediated by menin in the nervous system, we performed menin‐ChIP‐Seq, menin‐CUT&Tag and RNA‐Seq to screen and identify the downstream genes regulated by menin in the nervous system. We found that the transcription of *Foxg1*, a pathogenic gene associated with *FOXG1* syndrome,^[^ ^1^, ^4^ ^]^ significantly decreases under *Men1* deletion conditions. Menin typically interacts with other transcription factors to coordinate gene transcription. Through a menin‐binding protein interactome proteomics assay, we successfully identified Atrx (alpha‐thalassemia mental retardation X‐linked) as a menin‐associated protein. The Atrx protein is a member of SWI/SNF‐like chromatin remodeling protein, and its mutations are associated with X‐linked syndromes that exhibit cognitive disabilities.^[^ ^5^ ^]^ Menin collaborates with Atrx to regulate the transcription of *Foxg1*. The restoration of *Foxg1* expression in *Men1*‐CcKO mouse brain mitigated the neuronal impairments and ASD‐like behaviors. Our findings demonstrate the crucial role of menin in neuronal development and highlight its significance for FOXG1 syndrome, underscoring the need for further investigation to facilitate therapeutic development.

Epigenetic regulation is tightly associated with neurodevelopmental disorders such as autism disorder spectrum (ASD).^[^ ^6^ ^]^ Histone lysine methyltransferases play an important role in histone methylation and are commonly referred to as “writers” for catalyzing histone methylation.^[^ ^7^ ^]^ Mixed lineage leukemia proteins‐1 (MLL1) and Mixed lineage leukemia proteins‐2 (MLL2) are crucial members of histone methyltransferase family. MLL1/2 typically interact with multiple endocrine neoplasia type 1 (menin, gene for *Men1*) to increase the expression levels of the downstream target genes such as HOX, MEIS1, and EZH2.^[^ ^8^ ^]^ Histone methylation, mediated by MLL1 and MLL2, plays pivotal roles in the development of nervous system. Mice with neuronal‐specific *Mll1* or *Mll2* deletions exhibit reduced synaptic plasticity and impaired working memory formation by regulating important neuronal development genes.^[^ ^9^ ^]^ However, the function of menin in the central nervous system are largely unknown.

Menin is encoded by the multiple endocrine neoplasia type 1 (*MEN1)* in humans (*Men1* in mice). It serves as a crucial transcriptional activator for MLL1 and MLL2, thereby exerting significant influence on histone methylation modification.^[^ ^10^ ^]^ *MEN1* syndrome is a autosomal dominant disorder characterized by multiple tumorigenesis in endocrine organs, caused by loss‐of‐function mutations of *MEN1*.^[^ ^11^ ^]^ Genetic deletion of *Men1* induces embryonic lethality, including impairments in the nervous system development, by regulating a large number of developmental genes.^[^ ^12^ ^]^ However, the underlying mechanism of menin in nerve development is not well understood. Through multifaceted transcriptome analyses specifically in neurons, we found that the transactivation of *Foxg1* is dramatically decreased in the absence of *Men1*, and which is attributed to non‐menin‐Mll1/2 mediated H3K4me3 modification.

## 2 Results

### 2.1 Menin Binds to the *Foxg1* Promoter Locus and Promotes its Transcription and Expression

**Figure 1:**
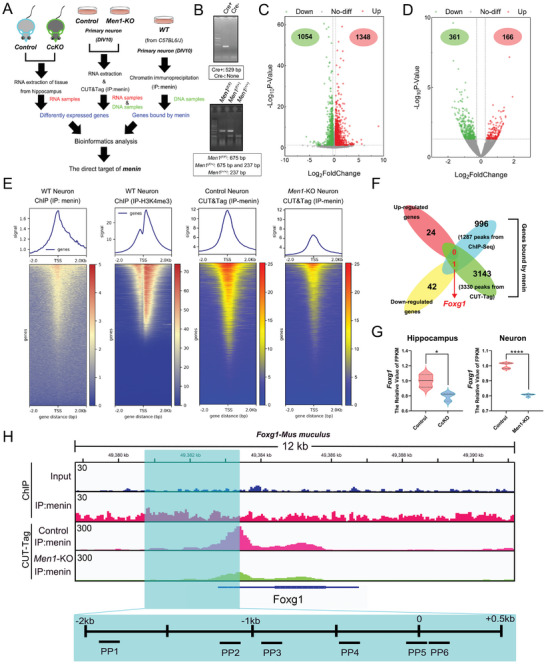
Menin binds to the promoter region of *Foxg1* and regulates the expression of *Foxg1*. A) Schematic of the experimental strategy used to identify the direct target of menin from multiple‐omics data. The RNA‐Seq analysis was performed on three independent biological replicates of mouse hippocampus and four independent biological replicates of primary neurons, while ChIP‐Seq or CUT&Tag experiments were limited to a single trial. B) Representative genotyping results are shown for *Men1* WT (*Men1^(+/+)^*), heterozygous (*Men1^(F/+)^*), homozygous (*Men1^(F/F)^*), as well as two Cre transgenic mice with expected PCR product size. C) Volcano plot showing differential expressed genes between *Men1‐*KO neurons and Control neurons (*p *< 0.05 and |Log_2_Fold change|>0.2630), There are 1348 up‐regulated genes and 1054 down‐regulated genes in *Men1*‐KO neurons. D) Another Volcano plot demonstrates differential expressed genes between CcKO mouse hippocampus and Control mouse hippocampus (*p *< 0.05 and |Log_2_Fold change|>0.2630), There are 166 up‐regulated genes and 361 down‐regulated genes in the hippocampus of *Men1*‐CcKO mice. E) Coverage profiles show the signal of menin and H3K4me3 in DIV10 primary neurons from ChIP‐Seq data, along with a heatmap displaying menin‐associated peaks centered at TSS, similar to the H3K4me3. And coverage profiles depict the signal of menin in *Men1*‐KO neurons compared to controls using CUT&Tag dataset. F) Venn diagram showing the overlap between Up‐regulated genes (vs. the control group) in *Men1*‐KO neurons and the hippocampus of *Men1*‐CcKO mice (*n* = 24 DEGs), Down‐regulated genes (vs. the control group) in *Men1*‐KO neurons and the hippocampus of *Men1*‐CcKO mice (*n* = 42 DEGs), respectively. *Foxg1* was further identified by intersecting the DEGs from RNA‐seq with the genes bound by menin in ChIP‐Seq/CUT&Tag analyses. G) The expression changes of *Foxg1* were examined in mouse hippocampus and primary neurons. FPKM value of *Foxg1* from different samples were normalized to the control group (*n* = 3 hippocampal samples per group and *n* = 4 primary neuronal samples per group). H) The IGV diagram showing menin binding position on the *Foxg1* gene from ChIP‐Seq and CUT&Tag data performed in primary neurons. A significant number of binding signals on the *Foxg1* TSS by menin are observed. And six ChIP‐PCR primer pairs were designed within a ≈2500 bps fragment (near the TSS) to validate the data (Boxed with the blue rectangle). (All mice were males. Data is represented by mean ±SEM. ****p < 0.0001 and *p < 0.05 indicate significance between the two indicated groups. ***G***: unpaired t‐test.).

To explore the molecular pathways mediated by menin, we conducted RNA‐Seq and ChIP‐Seq analyses of menin from hippocampal tissues or primary neurons with *Men1* deletion. The *Men1* ^(F/F)^ mice (Control) were bred with CaMK2α‐Cre or Nestin‐Cre mice to generate CaMK2α‐Cre;*Men1* ^(F/F)^ conditional knockout (*Men1*‐CcKO) mice or Nestin‐Cre;*Men1* ^(F/F)^ conditional knockout (*Men1*‐NcKO) mice, respectively.^[^ ^13^ ^]^ RNA sequencing (RNA‐seq) was performed on hippocampus tissues (from *Men1*‐CcKO mice and their littermate controls) and primary neurons (from *Men1*‐NcKO mice and their littermate controls) (**Figure** **1**A,B). A total of 2402 and 527 differentially expressed genes (DEGs) (|log2FC|>0.263, *p* < 0.05) were identified in primary neurons and hippocampus, respectively, when comparing the control and *Men1*‐knockout groups (Figure 1C,D; and Data S1 and S2, Supporting Information). The DEGs found in primary neurons and hippocampus were subjected to overlapping analysis, revealing that 66 DEGs changed in the same way, with 24 genes being down‐regulated and 42 genes being up‐regulated (Figure S1A, Supporting Information) (Data S3, Supporting Information). The subsequent analysis of these 66 DEGs using Gene Ontology (GO) and Kyoto Encyclopedia of Genes and Genomes (KEGG) revealed that menin‐mediated genes are closely related to neurodevelopmental and neuropeptide signaling pathways. (Figure S1B,C, Supporting Information) (boxed in red). Using String v10 and Cytoscape analysis, we found that the protein‐protein interaction (PPI) network of these core genes clusters in neurodevelopment, which includes *Foxg1* (Figure S1D, Supporting Information).

Menin, an essential component of histone methyltransferases complex, is capable of regulating H3K4me3 modification to transactivate gene expression.^[^ ^14^ ^]^ However, the menin‐H3K4me3 mediated genes transcription has been little studied in the nervous system. To investigate this, menin and H3K4me3 ChIP‐seq analyses were conducted in primary neurons (Figure 1A). The binding signal of menin and H3K4me3 was notably enriched proximal to transcription start sites (TSS). Furthermore, CUT&Tag is an improved version of ChIP‐seq that produces higher quality results with lower noise. Consequently, it was selected for further analysis of menin‐binding peaks and genes.^[^ ^15^ ^]^ By conducing CUT&Tag analysis, we further found that the menin binding signal was reduced near the TSS in *Men1*‐KO neurons (Figure 1E).

**Figure 2:**
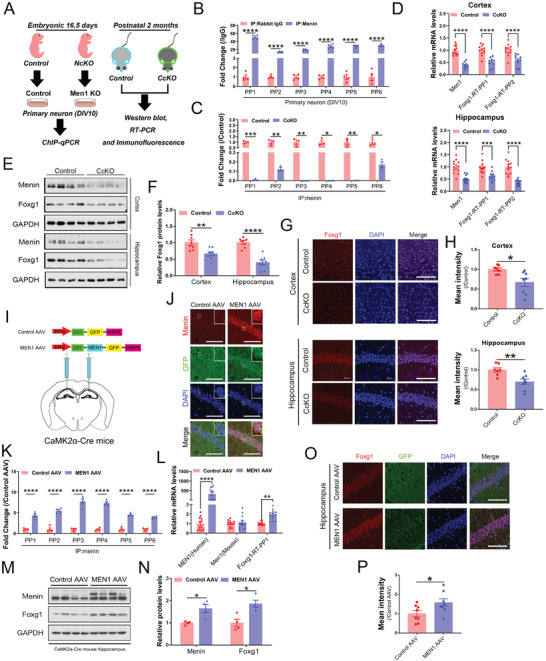
Menin transcriptionally regulates the expression of *Foxg1*. A) Schematic of the experimental strategy used to be verify the menin‐Foxg1 axis in vivo and in vitro. B) Six independent ChIP assays were performed in primary neurons to confirm menin binding to the *Foxg1* promoter (*n* = 4 independent experiments). C) The abundance of menin‐binding *Foxg1* promoter was compared between *Men1*‐CcKO mouse hippocampus and control. (*n* = 4 independent experiments). D) *Men1* and *Foxg1* mRNA levels were detected in *Men1*‐CcKO of mouse brain, including cortex and hippocampus (The cortical sample size was 12 in the control group and 10 in the *Men1*‐CcKO group; The hippocampal sample size was 12 in the control group and 12 in the *Men1*‐CcKO group). E) Menin and Foxg1 protein levels were measured by western blotting in *Men1*‐CcKO of mouse brain, GAPDH as internal control (The cortical sample size was 8 from 4 mice in the Control group and 8 from 4 mice in the *Men1*‐CcKO group; The hippocampal sample size was 8 from 4 mice in the control group and 7 from 4 mice in the *Men1*‐CcKO group). F) The diagram represents the statistics of the corresponding gray values in (E). G) Representative cortical (above) and hippocampal (below) brain sections from Control and *Men1*‐CcKO mice stained with Foxg1 (Red) antibody. Section was counterstained with DAPI (blue). Scale bar, 100 µm. (Control group: *n* = 6 slices from 3 mice; *Men1*‐CcKO group: *n* = 6 slices from 3 mice. Data was collected from cortex and hippocampus specimens). H) The corresponding statistical results for (G) are presented. I) Diagram of CaMK2α‐Cre mice were injected with AAV‐EF1α‐DIO‐MEN1‐GFP (*MEN1* AAV) into hippocampus to construct a *MEN1* overexpression model. J) Representative immunofluorescence image showing *MEN1* AAV and Control AAV. The red channel is menin, while green indicated GFP. Scale bar, 200 µm. High magnification insets are provided at the top of each image. (Control AAV: *n* = 3 slices from 3 mice and *MEN1* AAV: *n* = 3 slices from 3 mice). K) AAV‐mediated human *MEN1* overexpression in the hippocampus significantly enhanced the enrichment of menin binding to the *Foxg1* promoter (*n* = 4 independent experiments). L) *Men1* and *Foxg1* mRNA levels were detected in *MEN1*‐overexpressing hippocampus. The mRNA level of human‐derived *MEN1*, but not mice‐derived *Men1*, showed a significant increase (Control AAV group: *n* = 10 samples from 10 mice; *MEN1* AAV group: *n* = 12 sample from 12 mice). M) Menin and Foxg1 protein levels were measured by western blotting in *MEN1* overexpressing hippocampus, GAPDH as an internal control (Control group: *n* = 4 samples from 4 mice; *Men1*‐CcKO group: *n* = 4 samples from 4 mice). N) The diagram represents the statistics of the corresponding gray values in (M). O) Representative images of Foxg1 immunofluorescence were obtained from the *MEN1* AAV group and Control AAV group. The red channel represents Foxg1 and green is GFP. Scale bar, 200 µm (Control AAV group: *n* = 6 slices from 3 mice; *MEN1* AAV group: *n* = 6 slices from 3 mice.). P) The corresponding statistical results for (O) are shown. (All mice were males. Data is represented by mean ± SEM. ****p < 0.0001, ***p < 0.001, **p < 0.01, and *p < 0.05 indicate significance between the two indicated groups. ***B, C, D, F, K, L, N***: Two‐way ANOVA with Tukey post hoc test, ***H, P***: unpaired t‐test.).

A total of 2449 menin‐associated peaks were detected in the ChIP‐seq analysis, with 1287 peaks are distributed in the vicinity of 1000 genes. In the CUT&Tag analysis, a set of 3143 menin‐binding genes were identified from a pool of 3330 unique peaks (Data S4 and S5, Supporting Information). The analyses of GO and KEGG pathways for these ChIP binding genes revealed a high degree of clustering in neuronal development and maturation (Figure S1E, Supporting Information). As previously reported,^[^ ^16^ ^]^ menin predominantly occupies positions in intronic and intergenic regions (menin peaks: *n* = 1287, 43.5% in introns and 44.5% in intergenic regions) (Figure S1F, Supporting Information). Neuronal menin DNA binding motifs are also enriched, particularly the one characterized as “CAGCTG” (Figure S1G, Supporting Information). We performed integrated analysis by combining and overlapping ChIP‐seq, CUT&Tag and two RNA‐seq datasets. In the intersection analyses, only one gene, *Foxg1*, was observed significantly changed (Figure 1F). The decreasing of *Foxg1* was confirmed in menin deleted hippocampus and primary neurons (Figure 1G). The DNA binding peaks identified from the reading density of the ChIP‐Seq and CUT&Tag indicate that menin binds to the *Foxg1* gene, particularly in proximity to its transcription start site, as shown in CUT&Tag. Six primer pairs were designed based on the sequence near the transcriptional start site (TSS) for following validation (Figure 1H, boxed with the blue rectangle). **Figure** **2**A–C shows that all primers can bind to the *Foxg1* promoter region, as indicated by ChIP‐PCR experiments and these binding significantly decreased in *Men1* deleted primary culture neurons. Gel electrophoresis further demonstrated that menin is recruited to the *Foxg1* promoter in primary neurons and hippocampus tissues (Figure S2A,B, Supporting Information). A *Foxg1* promoter of approximately 2500 bps (Figure S2C, Supporting Information) was synthesized and fused to a luciferase reporter. Subsequently, this plasmid was then transfected along with control vector into both WT and *Men1* knockout primary neurons. The *Men1* deletion significantly decreased the activity of the *Foxg1* promoter‐driven luciferase in primary neurons (Figure S2D, Supporting Information).

### 2.2 Menin Transcriptionally Regulates the Expression of *Foxg1*

The above data suggests that menin may directly regulate the expression of *Foxg1*. By RT‐PCR, western blotting and immunostaining, we found a significantly decreased in both mRNA and protein levels of Foxg1 in the hippocampus and cortex of *Men1*‐CcKO mouse brains compared to the control (Figure 2D–H). To further investigate the regulation of menin on *Foxg1*, menin overexpression was conducted by injecting AAV‐EF1α‐DIO‐*MEN1* (*MEN1* AAV) into the hippocampus of CaMK2α‐Cre mice (Figure 2I). The expression of menin in the CA1 region was confirmed by immunostaining (Figure 2J). As anticipated, the binding of menin on the *Foxg1* promoter region was dramatically increased in the menin‐overexpressed hippocampus compared to control, as measured by menin‐ChIP‐PCR (Figure 2K). Concomitant with these findings, the mRNA and protein levels of Foxg1 were also increased by menin overexpression measured by RT‐PCR (Figure 2L), western blotting (Figure 2M,N) and immunostaining (Figure 2O,P).

To investigate whether the transcription regulation of menin on *Foxg1* is dependent on H3K4me3 modification, we performed an H3K4me3 ChIP assay in primary neurons and hippocampus tissues. Notably, two distinct primer pairs (PP1&PP2) targeting the *Foxg1* promoter locus both indicate the robust H3K4me3 at the *Foxg1* promoter in WT neurons and control hippocampus. Conversely, H3K4me3 occupancy at the *Foxg1* promoter locus dramatically decreased in *Men1*‐knockout neurons and hippocampus (Figure S2E,F, Supporting Information). Vice versa, extra menin expression increased a higher density of H3K4me3 on the *Foxg1* promoter was observed (Figure S2G, Supporting Information). Taken together, these results indicate that *Foxg1*, a causative gene of *Foxg1* syndrome, is regulated through modulation of histone H3 lysine 4 trimethylation by menin.

### 2.3 Deletion or Overexpression of Menin in Excitatory Neurons both Induces ASD‐like Behaviors in Mice

**Figure 3:**
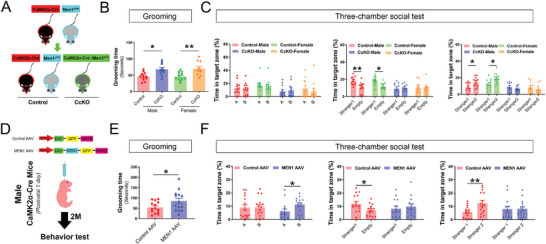
Excitatory neuron‐specific deletion or overexpression of menin both induce mice exhibiting ASD‐like behaviors. A) Establishment of the *Men1*‐CcKO transgenic model. B) Grooming tests were used to assess compulsive, rigid, repetitive behaviors in the four groups of mice. There were no significant differences in grooming time between males and females (Control‐male group: *n* = 12; Control‐female group: *n* = 12; *Men1*‐CcKO‐male group: *n* = 10; *Men1*‐CcKO‐female group: *n* = 10). C) Three‐chamber social tests were employed to analyze the social behavior in mice, including adaptive exploration stage (Left), social novelty tests (Middle) and social memory tests (Right) (Control‐male group: *n* = 12; Control‐female group: *n* = 12; *Men1*‐CcKO‐male group: *n* = 10; *Men1*‐CcKO‐female group: *n* = 10). D) The lateral ventricles of neonatal CaMK2α‐Cre male mice were intracerebroventricularly injected with Control AAV or *MEN1* AAV on postnatal day 0 (P0) to generate a neuron‐specific *MEN1*‐overexpressing model. E) Statistics of grooming time within 10 min under the free exploration state (Control AAV group: *n* = 15; *MEN1* AAV group: *n* = 14, all mice were males). F) Three‐chamber social experiments were employed to analyze the social behavior in the two groups of mice, including adaptive exploration stage (left), social novelty test (middle), and social memory test (right) (Control AAV group: *n* = 15; *MEN1* AAV group: *n* = 14, all mice were males). (Data is represented by mean ±SEM. *p < 0.05 and **p < 0.01 indicate significance between the two indicated groups. ***B***: one‐way ANOVA with Tukey post hoc test. ***C, F***: two‐way ANOVA with Tukey post hoc test. ***E***: unpaired t‐test.).

Patients with *FOXG1* syndrome present with mental retardation, lack of social ability, and intellectual disabilities.^[^ ^17^ ^]^ Both male and female *Men1‐*CcKO mice as well as control mice were subjected to behavior tests. Notably, these mice showed social deficits, increased stereotyped repetitive behaviors and cognitive deficits (**Figure** **3**A–C; Figure S3A, Supporting Information). There were no differences in locomotor activity were observed between the two groups based on rotarod assays (Figure S3B, Supporting Information). Furthermore, *Men1*‐deficient mice also exhibited impairments in hind limb clasping, as well as difficulties in the nest building and marble‐burying behaviors (Figure S3C–E, Supporting Information).

Another notable feature of *FOXG1* syndrome is its bidirectional dose effect on the causative gene. Both deletion and overexpression can lead to the emergence of ASD‐like behaviors in individuals.^[^ ^18^ ^]^ To investigate this further, we overexpressed menin by injecting *MEN1* AAV into the lateral ventricle of neonatal CaMK2α‐Cre mice, as depicted in Figure 2I and Figure S3J (Supporting Information) (Figure 3D). Overexpression of menin in excitatory neurons significantly induces ASD‐like behaviors in mice, accompanied by cognitive impairments assessed through grooming, social interaction, water maze and fear condition tests (Figure 3E,F; Figure S3F,G, Supporting Information), without affecting locomotor activity (Figure S3H,I, Supporting Information). These data indicate that enhanced the expression of excitatory neuronal menin is also associated with impairments in ASD‐like behaviors.

*Foxg1* is also expressed in inhibitory neurons and plays important function prompted us to investigate the involvement of menin‐*Foxg1* signaling pathway in interneurons for regulating ASD‐like behaviors.^[^ ^19^ ^]^ The conditional *Men1* interneuron‐specific deleted mice (*Men1*‐DcKO) were generated by crossing *Men1* ^F/F^ mice (Control) with Dlx5/6α‐Cre‐IRES‐GFP mice (Figure S3K, Supporting Information). Mice with *Men1* deficiency in inhibitory neurons (*Men1*‐DcKO) showed no significant changes in these phenotypes when compared to wild‐type animals (Figure S3L–N, Supporting Information). These findings suggest that the deficiency of Men1 in excitatory neurons, rather than inhibitory neurons, is associated with ASD‐like behaviors.

### 2.4 *Atrx* Knockdown Causes Autistic Behaviors and Contributes to the Transcription Regulation of *Foxg1* by Menin‐Mediated Histone H3 Lysine 4 Trimethylation

Since MLLs maintain gene expression through interaction with menin,^[^ ^10a^ ^]^ we examined whether menin‐MLL is involved in the regulation of *Foxg1* transcription. Primary neurons were subjected to treatment with menin‐MLL inhibitors MI‐2 and MI‐3, which specifically target the interaction between menin and MLLs.^[^ ^10b^ ^]^ However, we observed no significant changes in *Foxg1* expression following treatment with either MI‐2 or MI‐3 (Figure S4A–D, Supporting Information). As the positive control, the mRNA level of *Stab*2 was decreased (Figure S4E, Supporting Information).^[^ ^9a^ ^]^ This data suggests that other factors are required for menin to regulate the transcription of *Foxg1*.

**Figure 4:**
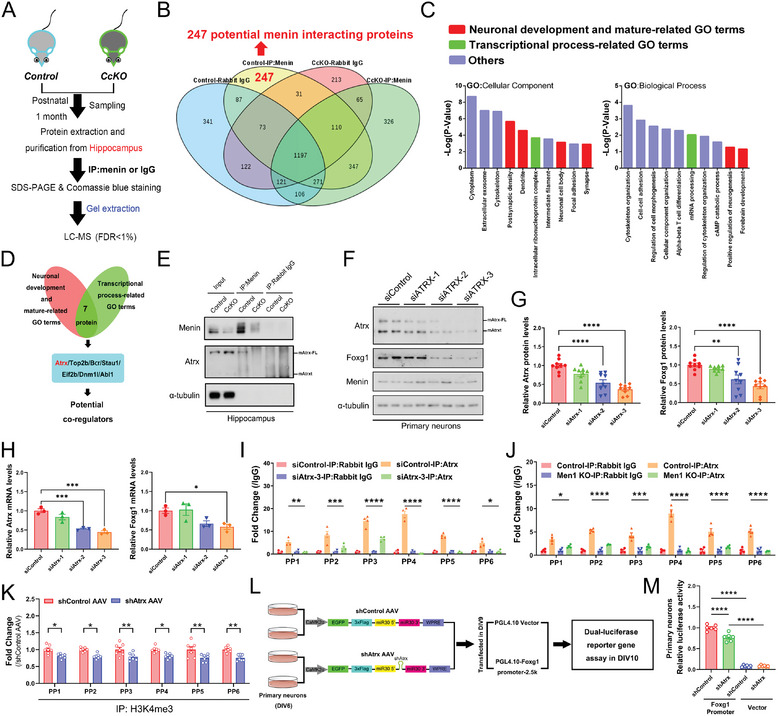
*Atrx* knockdown causes autistic behaviors and contributes to the transcription regulation of *Foxg1* by menin. A) The experimental strategy used to identify the interacting proteins of menin in control and *Men1*‐CcKO mouse brain is schematically depicted. Hippocampus tissues were obtained from 1‐month‐old *Men1*‐CcKO mice and littermate control mice (*Men1^(F/F)^* mice). The menin‐IP‐MS experiment was conducted in two independent trials. B) Interaction analysis among four protein sets revealed menin‐specific interacting proteins and a total of 247 menin‐specific interacting proteins were selected. C) The top 10 enriched cellular compound and biological process contributing to menin function were determined by menin‐specific interacting proteins. Red columns represent neuronal development and mature‐related GO terms, and green columns represent transcriptional process‐related GO terms, while blue columns represent other terms. D) 7 potential factors were screened from menin‐interacting proteins involved in both neuronal development and transcriptional regulation. Atrx may be an important factor involved in the regulation of menin‐Foxg1. E) Interactions between Atrx and menin were determined in control and *Men1*‐CcKO mouse brains. Hippocampus tissues came from 1‐month‐old *Men1*‐CcKO mice and littermate control mice (*Men1^(F/F)^* mice) (*n* = 3 independent experiments). F) Primary neurons were transfected with three *siAtrx* sequence for 72 h, followed by Western blotting of Atrx, menin and Foxg1 protein levels. α‐tubulin was used as a loading control (*n* = 3 independent experiments). G) The corresponding statistical results for (F) are shown respectively. H) Primary neurons were transfected with three *siAtrx* sequence for 72 h, followed by real‐time PCR to detect the mRNA expression of *Atrx* and *Foxg1*. β‐actin was used as a loading control. (*n* = 3 samples from 3 mice per group). I) The enrichment of Atrx binds to *Foxg1* promoter after interfering with *Atrx* expression in primary neurons. (*n* = 4 independent experiments and 4 samples per group). J) The enrichment of Atrx binds to *Foxg1* promoter was compared between *Men1*‐KO and WT primary neurons (Primary neurons are obtained from E16.5 embryos *Men1*‐NcKO mice and littermate control mice). (*n* = 4 independent experiments and 4 samples per group). K) The signal intensity of H3K4me3 on the *Foxg1* promoter after interfering with *Atrx* expression in primary neurons. (*n* = 4 independent experiments and 7 samples per group). L) To assessment of Atrx‐mediated transactivation of *Foxg1*, transfecting primary neurons with luciferase reporter plasmids after interfering *Atrx* expression level. The same fragment of *siAtrx*‐3 was inserted into AAV‐CaMK2a‐EGFP‐3xFlag‐miR30shRNA‐WPRE. M) The luciferase activity of *Atrx*‐mediated transactivation of *Foxg1* was measured. (*n* = 6 samples per group). (All mice were males. Data is represented by mean ± SEM.*p < 0.05, **p < 0.01, ***p < 0.001, and ****p < 0.0001 indicate significance between the two indicated groups. ***G, H, M***: one‐way ANOVA with Tukey post hoc test. ***I, J, K***: two‐way ANOVA with Tukey post hoc test.).

To investigate the potential interacting proteins of menin associated with the transcription regulation of *Foxg1*, we performed menin‐immunoprecipitation (IP) and liquid chromatography‐tandem mass spectrometry (LC‐MS) in hippocampus tissue from *Men1*‐CcKO mice and the controls (**Figure** **4**A). Label‐free LC‐MS/MS analysis identified 247 potential menin‐associated proteins (≥one unique peptide with an FDR < 1%) (Figure 4B) (Data S6, Supporting Information). These proteins were mostly enriched in neuronal development and mature GO terms, as well as transcriptional process‐related GO terms (Figure 4C). Notably, seven of these overlapping proteins are involved in neuronal development and serve as transcription factors (Figure 4D). Among the aforementioned seven candidates, ATRX is found to be associated with Alpha Thalassemia/Mental Retardation Syndrome X‐Linked, a rare congenital disorder with cognitive impairment. Another reason for selecting Atrx is that the ATRX‐DAXX‐menin complex plays a crucial role in neuroendocrine tumors.^[^ ^11^, ^20^ ^]^ The immunoprecipitation (IP) analysis in hippocampus tissue further confirmed that Atrx binds to menin in vivo (Figure 4E). It is noteworthy that *Atrx* gene encodes two main splicing isoforms: the full length Atrx (Atrx‐FL) and a truncated isoform (Atrxt).^[^ ^21^ ^]^ *Men1* deletion resulted in decreased expression levels of Foxg1 but not that of Atrx (Figure S4F,G, Supporting Information). On the other hand, the knockdown of *Atrx* by small interfering RNAs reduced the mRNA and protein levels of Foxg1 (Figure 4F–H). This reduction may be due to the decreased abundance of Atrx binding to the *Foxg1* promoter (Figure 4I). Additionally, the signal intensity of Atrx‐binding to the *Foxg1* promoter was significantly reduced in *Men1*‐knockout neurons (Figure 4J). *Atrx* silencing reduced the abundance of H3K4me3 marks on the *Foxg1* promoter in neurons (Figure 4K). By luciferase assay, we also found that the transcriptional activity of *Foxg1* dramatically decreased when *Atrx* was knockdown by *shAtrx* virus (Figure 4L,M).

**Figure 5:**
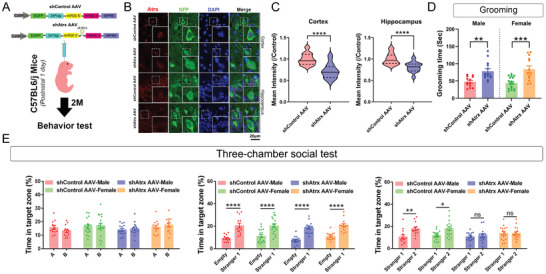
Partially *Atrx* Deletion causes autistic like behaviors. A) The lateral ventricles of neonatal C57BL6/J mice were intracerebroventricularly injected with shControl AAV or *shAtrx* AAV on postnatal day 1 (P1) to generate an excitatory neuron‐specific *Atrx* deficiency mouse model. B) The injection of shControl AAV or *shAtrx* AAV was performed in newborn C57 mouse bilateral ventricles. Representative confocal images of brain sections stained with Atrx (red) antibody. Section was counterstained with DAPI (blue). The green channel represents GFP. Scale bar, 20 µm. Insets marked by white boxes show enlarged cells. C) The corresponding statistical results of the fluorescence of Atrx in (B) were shown respectively. The left graph shows cortical statistics, while the right graph shows hippocampal statistics. (*n* = 30 GFP‐positive neurons. shControl AAV group: *n* = 6 slices from 3 mice; sh*Atrx* AAV group: *n* = 6 slices from 3 mice, all mice were males). D) Grooming tests were used to assess compulsive, rigid, repetitive behaviors in the four groups. (shControl AAV‐male group: *n* = 13; shControl AAV‐female group: *n* = 17; *shAtrx* AAV‐male group: *n* = 14; *shAtrx* AAV‐female group: *n* = 13). E) Three‐chamber social tests were employed to analyze social behavior in mice, including adaptive exploration stage (left), social novelty tests (middle) and social memory tests (right) (shControl AAV‐male group: *n* = 13; shControl AAV‐female group: *n* = 17; *shAtrx* AAV‐male group: *n* = 14; *shAtrx* AAV‐female group: *n* = 13).(Data is represented by mean ± SEM. ns: not significant, *p < 0.05, **p < 0.01, ***p < 0.001, and ****p < 0.0001 indicate significance between the two indicated groups. ***D***: one‐way ANOVA with Tukey post hoc test. ***E***: two‐way ANOVA with Tukey post hoc test. ***C***: unpaired t‐test).

Subsequently, the knockdown of *Atrx* in excitatory neurons by intraventricular injecting *shAtrx* and control virus mediated by CaMK2α promoter into wild‐type neonatal mice (Figure S4H, Supporting Information). After 2 months, these mice were subjected to comprehensive behavioral and histological analyses (**Figure** **5**A). The Atrx protein level was significantly decreased by *shAtrx* AAV infection, accompanied by down‐regulation of *Foxg1* (Figure 5B,C; Figure S4I–L, Supporting Information). Behavior tests indicate that knockdown of excitatory neuronal *Atrx* significantly induces mice to exhibited repeating grooming behaviors (Figure 5D), impaired social memory (Figure 5E), and cognitive deficits (Figure S4M, Supporting Information). In summary, Atrx, a novel potential target of ASD, governs *Foxg1* transcriptional regulation by menin‐mediated histone H3 lysine 4 trimethylation.

### 2.5 Restoring *Foxg1* Expression in *Men1*‐CcKO Mice Relieves ASD‐Like Behaviors and Synaptic Deficits

**Figure 6:**
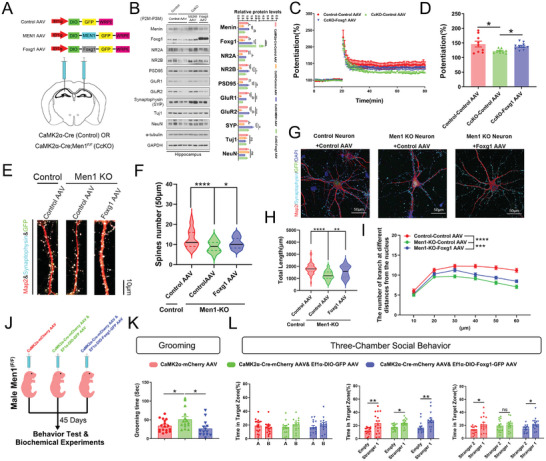
Foxg1 alleviates ASD‐like behaviors and synaptic dysfunction caused by *Men1* deficiency. A) The *Men1*‐CcKO mice and littermates were injected with AAV‐EF1α‐DIO‐GFP (Control AAV), AAV‐EF1α‐DIO‐*MEN1*‐GFP (*MEN1* AAV), and AAV‐EF1α‐DIO‐*Foxg1*‐T2A‐GFP (*Foxg1* AAV) into hippocampus respectively. B) Menin, Foxg1, NR2A, NR2B, PSD95, GluR1, GluR2, Synaptophysin (SYP), Tuj1, NeuN, and internal control proteins (α‐tubulin and GAPDH) were detected in different mouse hippocampal lysates using SDS‐PAGE electrophoresis. The corresponding statistics are shown on the right side. (*n* = 4 samples from 4 mice per group). C) LTP recordings of brain slices from litter‐ and age‐matched control and *Men1*‐CcKO mice that were injected with the indicated AAV viruses in panel A. D) The field excitatory postsynaptic potential (fEPSP) potentiation was quantified during the last 10 min of recording (Control‐Control AAV: *n* = 9 slices from 4 mice; *Men1*‐CcKO‐Control AAV: *n* = 9 slices from 4 mice; *Men1*‐CcKO‐*Foxg1* AAV: *n* = 10 slices from 5 mice). E) Primary neurons from Control and *Men1*‐KO mice were cultured in vitro and infected with AAV‐EF1α‐DIO‐GFP (Control AAV) and AAV‐EF1α‐DIO‐*Foxg1*‐GFP (*Foxg1* AAV) on DIV 3. Confocal projection images of GFP‐positive, Map2 (Red) and Synaptophysin (White) stained neurons on DIV 12 are shown. Scale bar: 10 µm. (*n* = 60 dendrites in each condition). F) The corresponding statistical results for (E) are shown. G) Primary neurons from Control and *Men1*‐deficient mice were cultured in vitro and transduced with AAV‐EF1α‐DIO‐GFP and AAV‐EF1α‐DIO‐*Foxg1*‐GFP on DIV 3. Confocal projection images of Map2 (red) and Synaptophysin (baby blue) stained neurons on DIV 12 are shown. Scale bar, 50 µm. (*n* = 60 neurons in each condition). H) Summary of dendritic arborization. I) Summary of total length. J) Workflow of stereotactic AAV injection and rescue experiments. K) Grooming tests were used to assess the repetitive patterns of behavior in the three groups. L) Three chamber social tests were used to assess the social ability of the three groups, including adaptive exploration stage, social novelty and social memory (From left to right). (All mice were males. CaMK2α‐mCherry AAV: *n* = 15; CaMK2α‐Cre‐mCherry AAV& GFP AAV: *n* = 15; CaMK2α‐Cre‐mCherry AAV& *Foxg1* AAV: *n* = 15. Data is represented mean ± SEM. ns: not significant, *p < 0.05, **p < 0.01, ***p < 0.001, and ****p < 0.0001 indicate significance between the two indicated groups. ***D, G, I, L***: one‐way ANOVA with Turkey post hoc test. ***B, E***: two‐way ANOVA with Turkey post hoc test. ***K***: paired t‐test.).

Finally, we reinstated the expression of menin and Foxg1 by administering AAV‐EF1α‐DIO‐*MEN1*‐GFP (*MEN1* AAV) and AAV‐EF1α‐DIO‐*Foxg1*‐GFP (*Foxg1* AAV) virus, as well as AAV‐EF1α‐DIO‐GFP (Control AAV) into the bilateral CA1 region of the hippocampus in *Men1*‐CcKO mice and their littermates (**Figure** **6**A). The levels of synaptic proteins, including GluR1/2, NR2A/2B, synaptophysin and PSD95, were decreased in the hippocampus of *Men1*‐CcKO mice compared to controls. The expression of these proteins was normalized by restoring menin or Foxg1 (Figure 6B). The hippocampal long‐term potentiation (LTP) defects in *Men1*‐CcKO hippocampus were also attenuated by Foxg1 restoration (Figure 6C,D). Overexpression of *Foxg1* also ameliorated the neuronal development defects induced by menin deficiency, as it increased the synaptic number, the neuronal dendritic complexity and total dendritic length in *Men1*‐KO primary neurons (Figure 6E–I).

Using another strategy, we injected CaMK2α‐Cre AAV and AAV‐EF1α‐DIO‐*Foxg1*‐GFP into *Men1* ^F/F^ mice to simultaneously overexpress *Foxg1* and down‐regulate *Men1* in excitatory neurons (Figure 6J). The behavioral tests were subsequently performed 45 days after the injection. Immunostaining confirmed a decreased menin expression and an increased *Foxg1* expression in the cortex and hippocampus of the injected animals (Figure S5A,B, Supporting Information). Notably, overexpression of *Foxg1* partially rescued incremental repetitive behaviors (Figure 6K), social disorders (Figure 6L) and cognitive impairments (Figure S5C, Supporting Information) in neuronal menin knockdown mice. Therefore, restoring *Foxg1* expression can alleviate ASD‐like behaviors and synaptic dysfunction caused by *Men1* deletion.

## 3 Conclusion

Menin is a nuclear scaffold protein that participates in transcriptional regulation through histone modification, such as lysine trimethylation at H3K4 and H3K27.^[^ ^22^ ^]^ MLL1/2 belong to the trithorax group and specifically methylate histone H3K4 to produce the H3K4me3 modification. Deletion of *Mll1* or *Mll2* specifically in neurons in mice resulted in reduced synaptic plasticity and impaired working memory by regulating the expression of important genes involved in neuronal development.^[^ ^9^ ^]^ Menin also can coordinate with EZH2‐PRC2 to catalyze H3K27me3 to suppress the gene expression.^[^ ^23^ ^]^ Recently, menin has also been identified as a new H3K79me2 reader and is associated with the binding sites of H3K79me2 that span across their gene bodies.^[^ ^24^ ^]^ These findings indicate that menin plays a complex role in histone modifications. Menin is a multifunctional epigenetic regulator that interacts with various proteins to perform a variety of functions. Here, we found that menin associates with Atrx to regulate the transcription of *Foxg1* by H3K4me3.

Interestingly, the deletion or overexpression of menin both induces the mice exhibited autism‐like behaviors, suggesting that the expression level of menin is crucial for the neuronal functions. The bidirectional effects of menin also reflect its downstream gene *Foxg1*. Since both heterozygous *FOXG1* duplication or *FOXG1* deletion can lead to *FOXG1* syndrome.^[^ ^1^, ^25^ ^]^ Meanwhile, the bidirectional effect of menin on autism‐like behaviors is similar to that of another well‐known pathogenic gene in Rett syndrome: *MeCP2*. Loss of function of *MeCP2* induces the Rett syndrome phenotype, while gain of function promotes *MeCP2* duplication syndrome.^[^ ^26^ ^]^ Menin and MeCP2 are both important epigenetic regulators that play bidirectional roles in regulating gene expression.^[^ ^27^ ^]^

Furthermore, single‐nucleus RNA sequencing of cortical tissues from patients with ASD revealed that excitatory cortical neurons are the main cell types preferentially affected in ASD.^[^ ^28^ ^]^ In this study, we found that *Men1* deletion in excitatory neurons, but not inhibitory neurons, led to mice exhibiting autism‐like behaviors, which aligns with the aforementioned findings in ASD patients. We recently reported that *Men1* deletion in PV interneurons induced mice to exhibit depressive‐like behaviors.^[^ ^29^ ^]^ The effects of menin on interneurons, especially in different types of interneurons, deserve thorough investigations. The regulation of *FOXG1*‐associated transcription is crucial for neuronal development and synaptic plasticity.^[^ ^30^ ^]^ The recent studies have shown that Foxg1 plays a crucial role in regulating the specification of postmitotic neurons. Knocking out *Foxg1* has been found to result in deficits in neuronal projection.^[^ ^31^ ^]^ Overexpression of *Foxg1* attenuated the neuronal development defects induced by *Men1* deficiency.

The *ATRX* gene encodes a SWI/SNF‐like chromatin remodeling protein and is frequently mutated in various nervous system‐related tumors.^[^ ^32^ ^]^ Deletion of *Atrx* in excitatory neurons selectively leads to working memory deficits in male mice.^[^ ^20^, ^33^ ^]^ ATRX interacts with EZH2 to facilitate the deposition of H3K27me3.^[^ ^34^ ^]^ Furthermore, ATRX may be recruited to the heterochromatin histone mark, H3K9me3, either indirectly through interaction with HP1 or via recruitment of MeCP2,^[^ ^35^ ^]^ or directly by binding to the ADD domain to H3K9me3, which is located adjacent to unmethylated H3K4.^[^ ^36^ ^]^ However, administration of menin‐MLL inhibitors (MI‐2 and MI‐3) did not alter *Foxg1* expression, suggesting a potential mechanism that remains to be revealed. Our hypothesis suggests that Atrx recruits menin to bind to the transcriptional start region of *Foxg1*, mediating the regulation of *Foxg1* expression through a non‐Mll1/2 dependent process involving H3K4me3. This has prompted us to re‐evaluate the role of the Atrx complex.

Our work further highlights the coordination between menin and Atrx in mediating neuronal development. Dysfunction of these proteins can induce ASD‐like phenotypes in mice. Interestingly, it has been shown that Atrx directly interacts with Mecp2, and Mecp2 deficiency disrupts Atrx PCH localization.^[^ ^36a^ ^]^ In our experiments, knockdown of *Atrx* specifically in forebrain excitatory neurons in mice led to impaired social memory, increased repetitive behaviors and spatial memory impairment. Therefore, *Atrx* is a potential target of ASD‐associated virulence gene.

Previously, we have demonstrated that menin can regulate the activity of cyclin dependent kinase 5 (Cdk5) by mediating the expression of its activator, *p35*.^[^ ^37^ ^]^ The deficiency of *p35* and *Foxg1* has a similar impact on cognitive functions in mice. However, *Foxg1* dysregulation mainly causes ASD‐like behaviors, including social defects, increased repetitive behaviors, and sporadic epileptic encephalopathy.

The study has certain limitations. First, we created an unnatural model of ASD‐like behaviors in mice by deleting *Men1* specifically from excitatory neurons, which is distinct from human clinical cases. It will be necessary to explore menin‐FOXG1‐related ASD cases in the clinical population and elucidate the precise genetic causes of the symptoms. Second, numerous reports show that ATRX, menin and FOXG1 are all involved in the pathogenesis of cancer.^[^ ^38^ ^]^ Further investigations are needed to determine whether the ATRX‐menin‐FOXG1 axis is an important comorbid target in *FOXG1* syndrome and cancer. Thirdly, ATRX is a transcription repressor that may stabilize chromatin by inhibiting transcription or replication through the DNA damage pathway. However, in this study, Atrx was found to be involved in the transcriptional activation of *Foxg1* mediated by menin. Further research is required to fully comprehend the regulatory mechanisms. Additionally, our study demonstrated that knockdown of *Atrx* resulted in the down‐regulation of *Foxg1* and ASD‐like behaviors. The overexpression of *Atrx* should be performed to investigate whether the restoration of *Atrx* can rescue the physiological and behavioral change in *Men1*‐CcKO mice. However, the *Atrx* gene is too large to be accommodated in the current viral expression system. It is worth noting that the loss of function of *Atrx* is closely correlated with the ASD phenotype. We will investigate the effect of *Atrx* overexpression for an extended duration. In summary, our finding demonstrates that menin/Atrx regulate *Foxg1* transcription through H3K4me3 and are involved in the pathogenesis of *FOXG1* syndrome.

## 4 Experimental Section

### Ethics Statement

The study was approved by the laboratory animal center at Xiamen University, and all experimental procedures involved were performed according to protocols approved by the Institutional Animal Care and Use Committee at Xiamen University. The approval number of the protocol is XMULAC20190144.

### Experimental Animals

All mice were reared on a 12/12 light/dark cycle. As described in the previous article.^[^ ^37^ ^]^ The *Men1* floxed mouse strain was crossed with CaMK2α‐Cre mice (JAX#:005359), Nestin‐Cre mice (JAX#:003771), and Dlx5/6α‐Cre‐IRES‐GFP(JAX #:023724) mice respectively.^[^ ^13^, ^39^ ^]^ Without extra emphasis, Mice aged between 2 to 4 months old were selected for the experiment. Litter‐ and age‐matched male mice were exclusively used for behavioral tests, electrophysiology, and biochemistry experiment to exclude interfering effects derived from estrogen.

### Newborn Mice were Injected with Adeno‐Associated Virus and Stereotactic Injection of Adeno‐Associated Virus in Adult Mice

pAAV‐EF1α‐DIO‐GFP‐WPRE (virus titer: 5.25 × 10^12^/ml), pAAV‐EF1α‐DIO‐MEN1 ‐GFP‐WPRE (virus titer: 6.29×10^12^/ml), pAAV‐EF1α‐DIO‐Foxg1‐T2A‐GFP‐WPRE (virus titer: 2.95 × 10^12^/ml) were purchased from BrainVTA (WuHan, China). Additionally, pAAV‐CaMK2α‐EGFP‐3xFlag‐miR30‐shRNA (Atrx)‐WPRE (virus titer: 5.14 × 10^12^/ml) and pAAV‐CaMK2α‐EGFP‐3xFlag‐miR30‐shRNA (NC)‐WPRE (virus titer: 8.53 × 10^12^/ml) were purchased from OBIO (ShangHai, China). All viral vectors were aliquoted and stored at −80 °C until used.

pAAV‐EF1α‐DIO‐GFP‐WPRE and pAAV‐EF1α‐DIO‐MEN1‐GFP‐WPRE were injected into the bilateral ventricles of newborn CaMK2a‐Cre mice respectively at the same virus titer (virus titer: 5 × 10^12^/ml, 1 ul, one side)^[^ ^40^ ^]^ to overexpress *MEN1* in excitatory neurons for behavioral assessment of the mice.

pAAV‐CaMK2α‐EGFP‐3xFlag‐miR30‐shRNA(Atrx)‐WPRE (virus titer: 5.14 × 10^12^/ml) and pAAV‐CaMK2α‐EGFP‐3xFlag‐miR30‐shRNA(NC)‐WPRE (virus titer: 8.53 × 10^12^/ml) were injected into the bilateral ventricles of newborn C57/BL6J mice respectively at the same virus titer (virus titer: 5 × 10^12^/ml, 1 ul, one side) to further evaluate ASD‐like behaviors in mice after the intervention of *Atrx* expression.

pAAV‐EF1α‐DIO‐GFP‐WPRE, pAAV‐EF1α‐DIO‐MEN1‐GFP‐WPRE, pAAV‐EF1α‐ DIO‐Foxg1‐T2A‐GFP‐WPRE, pAAV‐CaMK2a‐mCherry‐T2A‐Cre were stereotactically injected into bilateral hippocampus of male *Men1^(F/F)^* mice at the same virus titer (virus titer: 2.5 × 1012/ml, 1ul, one side). The specific spatial coordinates were X = ±1.7, Y = ‐2.0, and Z = ‐1.7 mm. To confirm the overexpression of *MEN1* or *Foxg1* in mouse brains, mice were anesthetized and sacrificed 21 days after injection. Subsequently, brain tissues were rapidly removed and analyzed using histological immunofluorescence staining and western blotting.

### Primary Neurons Culture, Small Interfering RNA, Plasmids and Drug Experiments

A mixture of primary cortical and hippocampal neurons was dissected from timed‐pregnant females at E16.5. *Men1* knockout neurons were derived from *Men1*‐NcKO (Nestin‐Cre;*Men1* ^(F/F)^) mice, while Control neurons were derived from litter‐matched *Men1^(F/F)^* mice. Briefly, brains of pups with varying genotypes were dissected. The meninges were removed, and the tissue was dissociated by enzymatic digestion. Isolated primary neurons were plated on poly‐D‐lysine coated dishes, and cultured in Neurobasal medium supplemented with B27 (Gibco, 17504044), 1% penicillin/streptomycin (Invitrogen) and maintained in a 5% CO_2_ incubator at 37 °C.

*Atrx* Small interfering RNA (*siAtrx*) were customized from Tsingke Biotechnology Co., Ltd. On DIV6 primary neurons, several siRNA were transfected with Lipofectamine 3000 (Thermo fisher, L3000150). After 72 h, western blotting or chromatin immunoprecipitation (ChIP) was performed. On DIV10 primary neurons were treated with MI2 (MCE, HY‐15222) and MI3 (MCE, HY‐15223) at a concentration of 1 µm each. After 24 h, western blotting or RT‐PCR was performed. The pGL4.0‐Foxg1 promoter‐2.5 k plasmid and the pGL4.0[LUC2] plasmids were obtained from Tsingke (China, Beijing). In short, the fragment of *Foxg1* promoter‐2.5 K was synthesized, according to the Mouse Genome Informatics was inserted into the pGL4.0[LUC2] plasmid using sites‐directed insertion technique. The two plasmids were severally transfected with Lipofectamine 3000 (Thermo fisher, L3000150) into primary neurons on DIV10 after 3 day's treatment of *shAtrx* AAV or shControl AAV.

### Tissue and Cell Lysate Preparation, and Antibodies Used for Immunoblotting

Cortex and hippocampus samples were prepared from at least three pairs of male mouse brains, including Control and Men1‐CcKO mice. Proteins extracted from primary neurons or organs tissue lysates were analyzed using western blotting. The proteins were separated on SDS‐polyacrylamide gel electrophoresis and probed with specific antibodies, including menin (rabbit, 1:1000; Abcam, ab31902), Foxg1 (rabbit, 1:1,000; Abcam, ab196868), Atrx (rabbit, 1:1000, Bethyl Laboratories, A301‐045A‐T), PSD95 (rabbit, 1:1000; Cell signaling Tech., #2507), GluR1 (rabbit, 1:1000, Cell signaling Tech., #13185), GluR2 (rabbit, 1:1000, Cell signaling Tech., #13607), NR2A(rabbit, 1:500, Sangon Biotech, D160672), NR2B (rabbit, 1:500, Sangon Biotech, NO. D260673), Synaptophysin (rabbit, 1:000; Cell signaling Tech., #36406), Tuj1 (TUBB3) (mouse, 1:1000, Biolegend, # MMS‐435P), NeuN (mouse, 1:1000, Millipore, MAB377), β‐actin (mouse, 1:1,000; Cell Signaling Technology, #3700), GAPDH (mouse, 1:5000, Invitrogen, #MA5‐15738), α‐tubulin (mouse, 1:5000, abclonal, 1:5000, AC012). Goat‐anti‐mouse secondary antibodies and goat‐anti‐rabbit secondary antibody were purchased from Millipore (#AP132P, #AP124P). For quantification, densitometry measurements of each band (Image J) were first normalized to β‐actin or GAPDH or α‐tubulin, and averaged from at least three independent experiments. More details are provided in Data S7 (Supporting Information).

### Immunofluorescence Staining

Immunofluorescence staining was performed as previously described.^[^ ^37^ ^]^ Mouse brain sections or cultured cells were fixed in 4% paraformaldehyde and washed three times with PBS. Antigen retrieval was performed using citrate buffer (pH7.0), followed by permeabilization and blocking in PBS containing 0.5% Triton X‐100 and 10% normal goat serum at room temperature for 1 h. Brain sections or cultured cells were incubated with primary antibodies in the blocking buffer overnight at 4 °C, then probed with secondary antibodies in the same buffer for 1 h on the following day. Samples were washed and stained with DAPI. Images were acquired using a Nikon confocal microscope and a Zeiss LSM 880 Airyscan in the Core facility of Biomedical sciences at Xiamen University. Primary antibodies used for immunostaining include: menin (rabbit, 1:500; Abcam) GFP (mouse,1:1000; Santa Cruz), MAP2 (rabbit, 1:1000; Cell Signaling Technology), Tuj1(mouse,1:1000; Biolegend), Foxg1 (rabbit, 1:500; Abcam, ab196868), Atrx (rabbit, 1:200, Bethyl Laboratories, A301‐045A‐T) et al. 488/594 donkey anti‐mouse/rabbit secondary antibodies (1:1000) and Mounting Medium with DAPI was purchased from Invitrogen. Further information on the antibodies used can be found in Data S7 (Supporting Information).

### Co‐Immunoprecipitation

The mice were quickly killed by carbon dioxide and their brains were separated. Wash the brain twice with ice‐cold PBS and fully homogenize the tissue using the homogenate in IP buffer (Tris‐Hcl pH = 7.4 0.05m; Nacl 0.15 mm; EGTA 1 µm; EDTA 1 µm; Triton‐100 1%; NaPPi 0.223 g; NaVO3 1 µm; ddH2O Up to 200 ml). Then, gently pipette up and down on ice to lyse the brain for 2 h (10 times every 10 min). Centrifuge at 12,000 x g for 15 min at 4 °C and transfer all supernatant to a new tube. Measure the protein concentration and check the expression of menin by SDS‐PAGE/Immunoblotting analyses (This step is optional but highly recommended.). Normally 5 µg of Atrx antibodies (CST‐#14820) or 5 µg of menin antibodies (Bethyl‐A300‐105A) were used in the lysate. Incubate the tube overnight at 4 °C on a rotator. The next day, use a large‐orifice pipette tip to transfer an adequate amount of Protein G (Thermo‐10004D) into a 1.5 ml tube (20–30 µl per Sample). Then, preclear the Protein G 3–4 times using a magnetic frame and IP buffer. Resuspend the Protein G in ice‐cold IP buffer (≈20 µl X (sample number + 1)). Add 20 µl precleaned Protein G into the lysate. Incubate the tube on a rotator for 2–4 h at 4 °C. After co‐incubation, aspirate the supernatant to remove non‐binding proteins and wash the pellet by resuspending it in 1 ml ice‐cold IP buffer. Resuspend the Protein G‐bound immune complexes in 30–40 µl 1x loading buffer, boil for 5–10 min, and analyze them by SDS‐PAGE/Immunoblotting, mass spectrometry, or other methods. If the sample would be analyzed by SDS‐PAGE/Immunoblotting, avoid using antibodies developed in the same species as the antibody used for immunoprecipitation.

### Liquid Chromatography‐Tandem Mass Spectrometry (LC‐MS)

Hippocampus dissection was performed and the LC‐MS experiment was conducted in double‐blind conditions. Briefly, please refer to the Co‐IP procedure mentioned above, where Protein G‐bound immune complexes were analyzed by SDS‐PAGE/Immunoblotting. The glue was cut into small pieces with a size of 1.5 mm^3^ and placed in centrifuge tube (Volume ≤ 300 uL in each tube). The glue was washed with ultra‐pure water (DDW) for more than three times and then decolorized with a decolorizing solution [40% acetonitrile (ACN), 50 mm ammonium bicarbonate (ABC) in H_2_O] until it became colorless. The sample was finally stored in the decolorizing solution. Trypsin (Promega, V5111) was added at a ratio of 1:50 (trypsin: protein, m/m) and enzymolyzed overnight. The fractions were dried for LC‐MS/MS analysis using a Bruker timsTOF Pro instrument. The resulting raw files were imported into MaxQuant software for data interpretation and protein identification against the database. This experiment received significant support from the Core Facility of Biomedical Sciences at Xiamen University. The data was successfully submitted to ProteomicXchange via the PRIDE database, with project accession number: PXD042795.

### Chromatin Immunoprecipitation (ChIP)

ChIP procedures were performed followed the manufacturer's instructions (17‐295; Millipore). Briefly, primary neurons from different genotypes of mice were cultured for 8–12 days and fixed for 10 min at room temperature with media containing 1% formaldehyde and quenched with 125 mm glycine for 5 min. Fixed homogenates were washed twice using ice‐cold PBS containing protease inhibitors. Fixed nuclei were pelleted at 2,000 rpm for 4 min and re‐suspended in SDS Lysis Buffer (catalog #20‐163), where chromatin was sheared using a SCIENTZ ultrasonic apparatus set to 28% power for 14 cycles of a 4.5 s sonication and a 9.0 s resting stage on ice. The sonicated cell supernatant was diluted ten‐fold in ChIP dilution buffer (catalog #20‐153) and pre‐cleared using protein An Agarose/Salomon Sperm DNA (catalog #16‐157). Five percent of sample was removed before the immunoprecipitation (IP) for input measurements. ChIP was performed using 5 µg menin antibody (A300‐105A; Bethyl), 5 µg Atrx antibodies (CST‐#14820), 3ug H3K4me3 (17‐614; Millipore), or normal rabbit IgG (H2615; Santa Cruz) antibody incubated overnight, followed by enrichment using Protein A Sepharose beads for 4 h. Beads were washed three times with four different buffers (low‐salt immune complex wash buffer, high‐salt immune complex wash buffer, and LiCl immune complex wash buffer) and one wash with TE (50 mm Tris HCl, 10 mm EDTA). Chromatin was eluted by agitation at 65 °C for 20 min in TES (TE plus 1%SDS) and reverse cross‐linked overnight at 65 °C. Chromatin was subjected to RNase and Proteinase K treatment, followed by DNA purification by phenol chloroform extraction and ethanol precipitation. DNA pellets were re‐suspended in 10 mm Tris and subjected to qPCR (480; Roche). The qPCR signals were calculated using the following equation: ① The background value was obtained by subtracting the ΔCT value of Input from the ΔCT value of IP_IgG_, and the actual value was obtained by subtracting the ΔCT value of Input from the ΔCT value of IP_specific antibody_. ②The background value was normalized by the average value of biological duplicate samples from the same group (defined as normalized background values) and the actual value of specific antibody was normalized to its corresponding background value (referred to as normalized actual values). ③ The fold change was calculated by using the formula: Fold Change = 2 raise to the power of (‐ normalized actual values or normalized background values). Primer sequences used for ChIP‐qPCR in this study are summary in Data S7 (Supporting Information).

### Behavior Tests

*Morris Water Maze*: The water maze used in this study comprised a circular tank 120 cm in diameter with a platform filled with tap water at a temperature of 22 ± 2 °C. Different shapes were posted along the walls of the tank, which served as spatial reference cues. A camera was mounted above the maze to record the swimming traces in the water maze. During the acquisition trials, the platform was submerged 1–2 cm below the water surface, mice were place into the maze at one of four points (N, S, E, W) facing the wall of the tank. Mice could search for a platform for 60 s. If a mouse failed to find the platform, it was guided to the platform and maintained on the platform for 10 s. Four trials a day were conducted with at an intermission of 1 h minimum between trials. Escape latency which indicated spatial learning memory acquisition, was recorded for each trial. On day 7, the platform was removed and a probe test was conducted. The percentage time spent in each of the four quadrants and the number of target (platform) area crossings, mean speed, total distance was recorded.

*Fear Conditioning Test*: Experiments were carried out using a fear conditioning system (StartFear, Panlab Harvard Apparatus). The apparatus consisted of a test chamber (25 cm × 30 cm × 25 cm, H × W × L), where the ceiling, front and back of the chamber was transparent, and the floor was made of a removable grid of stainless‐steel rods (3.2 mm diameter, 4.7 mm apart). Automated fear conditioning software (FREEZING, Panlab, Harvard apparatus) was used to control foot shocks. Video recording within the chamber monitored the activity of the mice throughout the procedure. On training days, mice were placed in the center of the grid floor and allowed to explore the test chamber for 120 s. Next, three consecutive vocal cues were administered at 80 db maintained for 30 s, and foot shocks were administered at 0.5 mA for 2 s, and the resulting response were recorded as baseline responses. Animals were removed from the chamber and returned to their regular habitation. One day later, each mouse was re‐exposed to the chamber for 600 s without any stimulus to assess context‐associated fear conditioning. After 1 h, mice were placed in bright condition and left to explore the test chamber for 120 s. A vocal cue of 80 db maintained for 180 s was administered, and freezing time within a 300 s span was recorded during the cued test. Mouse activity on the first day was used for baseline comparison.

*Rotarod Test*: Mice were placed on a stationary rotarod (AccuRotor Rota Rod Tall Unit, 63 cm fall height, 30 mm diameter rotating dowel; Accuscan, Columbus, OH). The dowel was then accelerated at 60 rpm min^−1^, and the latency to fall (in seconds) was recorded. The procedure was repeated for 4 consecutive trials, which were averaged to give the daily latency to fall for each mouse. If an animal fell off the rotarod rapidly (e.g., due to inattention or slips), they were placed back on the rotarod for an additional trial, and the latency was not included in the average for the day. The entire procedure was repeated the following 2 days for a total of 3 days. In addition to the average latency across the 4 trials per day, the maximum latency to fall per day was also analyzed.

*Three‐Chamber Sociability Test*: The test apparatus consisted of three chambers: a middle chamber (40 × 20 cm^2^) and another two chambers (40 × 20 cm^2^) in which wire cages containing stranger mice were placed. For the sociability test, the test animal was placed in the middle chamber for 5 min to habituate. Then, a wire cage containing a novel juvenile male mouse was placed in one of the side chambers, and an empty wire cage was placed in the other. The test animal was placed in the middle chamber for 10 min. Lastly, two wire cages containing two novel juvenile male mice were placed on both sides. The test animal was placed in the middle chamber for 10 min. The time spent in each chamber and the time spent near the wire cages were analyzed using SMART v.3.0 software.

*Grooming Behaviors*: Grooming behaviors were measured as described previously. In brief, to measure basal grooming behavior, mice were video‐recorded in the dark phase for 15 min. The measured grooming behaviors included face wiping, scratching/rubbing of head and ears and full‐body grooming. After recording the behaviors, the experimenters were blinded to the genotypes and manually analyzed the videotapes.^[^ ^41^ ^]^

*Nest‐Building Test*: Mice received a fresh nestlet and a new standard housing cage with fresh bedding 1 h before the beginning of the dark phase. Nest status was assessed at the end of the dark phase. Nests were scored according to categories similar to a previously described protocol: untouched nestlet (0 points), partially shredded nestlet (1 point), shredded nestlet with beginning of a nest‐like structure (2 points), full cup‐like nest (3 points), and round enclosure (4 points). Then, the mice were transferred into a cage with nestlet for a 30 min assessment of the ability of utilizing the nesting material.^[^ ^42^ ^]^

*Marble Burying*: Place one mouse into a corner of the cage containing marbles, being careful to place the mouse on bedding as far from marbles as possible, and place the filter‐top cover on the cage. Withhold food and water during the test to allow mouse to remain in the cage undisturbed for 30 min. The remove the mouse and return it to its home cage after test completion, taking extreme care not to move or dislodge the marbles in the process of removing the subject from the cage. Task scorers (2–3 peoples) blind to the treatment conditions or genotype of the mouse being tested to count the number of marbles buried. Score a marble as buried if two‐thirds of its surface area was covered by bedding. Average scores for the number of marbles buried for each mouse. Retrieved all 20 marbles from the bedding and disposed of bedding.^[^ ^43^ ^]^ ⑨Open field test: To explore locomotion and spontaneous activity, mouse behavior was characterized as they freely explored an open‐field plastic chamber (40 cm width × 40 cm length × 30 cm height) using a video tracking system (Smart 3.0). Two months old mice were placed in this arena for 10 min., and total distance and time spent in the center region (20 cm × 20 cm) was recorded.

### RT‐PCR (Real‐Time PCR)

Total RNA was extracted from the tissues using Trizol (Invitrogen, Carlsbad, CA, USA) according to manual instruction. About 60 mg of tissue was ground into powder by liquid nitrogen in a 1.5 ml tube and then homogenized for 2 min before being rested horizontally for 15 min. The mixture was centrifuged for 10 min at 12,000 × g at 4 °C, then the supernatant was transferred into a new EP tube with 0.2 ml chloroform/isoamyl alcohol (5:1). The mixture was vigorously shaken for 15 s and then centrifuged at 12,000 × g for 10 min at 4 °C. After centrifugation, the upper aqueous phase containing RNA was transferred into a new tube with an equal volume of supernatant of isopropyl alcohol, then centrifuged at 12.000 × g for 20 min at 4 °C. After deserting the supernatant, the RNA pellet was washed three times with 1 ml 75% ethanol and subsequently centrifuged at 12,000 × g for 3 min at 4 °C to remove residual ethanol. The pellet was air‐dry for 5–10 min in the biosafety cabinet. Finally, 25µl∼40µl of DEPC‐treated water was added to dissolve the RNA. Subsequently, total RNA was qualified and quantified using a NanoDrop and Agilent 2100 bioanalyzer (Thermo Fisher Scientific, MA, USA). Total RNA was then reverse‐transcribed in a reaction volume of 10 ml by the ReverTra Ace qPCR RT Kit (FSQ‐101). cDNA was amplified by real‐time quantitative RT‐PCR using SYBR Green (Roche) reagent. Samples were assayed in triplicate and GAPDH or β‐actin was used as an internal control. Primer sequences used for qPCR in this study are summary in Data S7 (Supporting Information).

### RNA Sequencing

To evaluate gene expression differences between *Men1*‐CcKO and *Men1* ^F/F^ mice, single‐end RNA libraries were prepared from hippocampus of *Men1*‐CcKO male mice and the littermate *Men1* ^F/F^ male mice were sequenced using BGISEQ‐500 (BGI, Shenzhen, China) and three independent biological replicate samples were sequenced for each group. From above dataset gene expression profiles of hippocampus of mice were acquired. Clean reads were aligned against the Ensembl mice (mm10) genomes using HISAT2 software (version 2.0.4). Bowties2 was employed to clean reads alignment to reference sequences to calculate gene alignment (version 2.2.5). The mapped fragments were used to calculate the expression levels of different transcripts by RSEM (version 1.2.12). Reads count information directly from the files generated by RSEM using a R programming language script was taken as input of DESeq2 for differentially expressed genes identification. Differentially expressed genes defined from the pairwise comparisons had to satisfy two selection criteria, including i) fold change larger than 1.2 and ii) corresponding adjusted P value less than 0.05. Gene ontology and KEGG pathway enrichment analysis were performed using DAVID. The sequencing data were deposited in the NCBI Sequence Read Archive (SRA) database under the accession: PRJNA826677.

To evaluate gene expression differences between *Men1*‐KO and Control primary neurons, single‐end RNA libraries were prepared from *Men1*‐KO primary neurons and Control primary neurons which were then sequenced using BGI system (BGI, Shenzhen, China) and three independent biological replicate samples were sequenced for each group. The gene expression profiles of primary neurons were obtained from the above dataset. The sequencing data was filtered with SOAPnuke by 1) Removing reads containing sequencing adapter;^[^ ^44^ ^]^ 2) Removing reads whose low‐quality base ratio (base quality less than or equal to 15) was more than 20%; 3) Removing reads whose unknown base (‘N’ base) ratio was more than 5%, afterward clean reads were obtained and stored in FASTQ format. The subsequent analysis and data mining were performed on Dr. Tom Multi‐omics Data mining system. The sequencing data were deposited in the NCBI Sequence Read Archive (SRA) database under the accession: PRJNA980296.

### ChIP‐Sequencing

The ChIP DNA libraries of primary neurons were sequenced on BGISEQ‐500. Reads were aligned to mm10 using SOAP aligner (version 2.21t) software. After format conversion and sorting, duplicate reads were removed by rmdup tool from the SAM tools package. To identify significant binding site of menin, the mapped sequence reads were processed with MACS2 against their matching control samples and only peaks with P values <10^−5^ were kept for further analyses. A Perl script annotate Peaks.pl in HOMER package was used to associate ChIP peaks with nearby genes and visualization of the location and the shape of called peaks utilizing Integrative Genomics Viewer (IGV). Furthermore, all motifs were identified by MEME software (http://meme‐suite.org/). The sequencing was carried out by BGI Tech (Shenzhen, China). The sequencing data were deposited in the NCBI Sequence Read Archive (SRA) database under the accession: PRJNA826745 and PRJNA980398.

### CUT&Tag

CUT&Tag was performed with Hyperactive In Situ ChIP Library Prep Kit for lllumina (pG‐Tn5) (TD901, Vazyme Biotech) according to the manufacturer's instructions. In brief, primary neurons were collected and bounded to Concanavalin A‐coated beads. Subsequently, cells were resuspended in antibody buffer and incubated with primary antibodies against menin antibody (A300‐105A; Bethyl) and secondary antibodies in order. The samples were incubated with pA‐Tn5 transposase. Aftertransposon activation and tagmentation, DNA was isolated, amplified, and purified to construct library. The library for sequencing was constructed and VAHTS DNAClean Beads (N411, Vazyme Biotech) were used for purification steps. The library was quantified with VAHTS Library Quantification Kit for lllumina (VazymeBiotech) and sequenced on a lllumina novaseq 150PE. The sequencing was carried out by Novogene (Novogene, Beijing, China). The raw sequence data are available from GEO accession number: GSE261274.

### Dual Luciferase Reporter Activity

Dual‐luciferase reporter assay was performed with the Dual‐Luciferase Reporter Assay System (Promega, E1910). Briefly, primary neurons were infected with AAV (shControl AAV or *shAtrx* AAV) in DIV 6 for knockdown *Atrx*. After culturing until DIV 9, the PGL‐4.10 or PGL‐4.10‐*Foxg1* promoter‐2.5K plasmid was transfected with Lipofectamine 3000 (Thermo fisher, L3000008). 24 h later, the infected cell lysates were harvested, and relative luciferase activity was determined by normalizing the relative luciferase unit readout of the firefly luciferase to that of the Renilla luciferase activity measured by Varioskan Flash (Thermo fisher).

### Statistical Analysis

All data represent mean ± SEM. Statistical analyses were performed using GraphPad Prism 9, Clampfit, or SigmaStat 4 statistical software. For statistical significance, experiments with two groups were analyzed using two‐tailed Student's tests and one‐way ANOVA. Experiments with more than two groups were subjected to one‐way ANOVA and two‐way ANOVA (*p < 0.05, **p < 0.01, ***p < 0.001, ****p < 0.0001). Detailed statistical analysis methods for each comparison are indicated in the figure legends, including sample size and pre‐processing of data, etc.

## Conflict of Interest

The authors declare no conflict of interest.

## Author Contributions

K.Z. and L.L. contributed equally to this work. J.Z. conceptualized the study; K.Z., X.S., S.W., Y.S., and Y.X. prepared and maintained the mice; K.Z., L.L., Y.Z., and Y.C. designed and performed morphological analysis and biochemical assays; L.L., K.Z., Y.S., L.Z., and S.Y. performed behavior tests; X.S. performed electrophysiology experiments. K.Z., H.L., and J.Z. wrote the manuscript. Z.C. and T.Y. discussed and edited the manuscript. J.Z. supervised the project. All authors reviewed and gave final approval to the manuscript.

## Supporting information

Supporting Information

Supplemental Data 1

Supplemental Data 2

Supplemental Data 3

Supplemental Data 4

Supplemental Data 5

Supplemental Data 6

Supplemental Data 7

## Data Availability

The data that support the findings of this study are available in the supplementary material of this article.
