# Supplementary material for: Menin Deficiency Induces Autism‐Like Behaviors by Regulating Foxg1 Transcription and Participates in Foxg1‐Related Encephalopathy
Source: Adv Sci (Weinh). 2024 Apr 6;11(24):2307953. doi: 10.1002/advs.202307953 (PMC11200012; doi:10.1002/advs.202307953)
Supplement: Supplementary file 1 — Supporting Information [file ADVS-11-2307953-s003.pdf]

ADVANCED SCIENCE
Open Access

Supporting Information

for *Adv. Sci.*, DOI 10.1002/advs.202307953

Menin Deficiency Induces Autism-Like Behaviors by Regulating *Foxg1* Transcription and Participates in *Foxg1*-Related Encephalopathy

*Kai Zhuang, Lige Leng, Xiao Su, Shuzhong Wang, Yuemin Su, Yanbing Chen, Ziqi Yuan, Liu Zi, Jieyin Li, Wenting Xie, Sihan Yan, Yujun Xia, Han Wang, Huifang Li, Zhenyi Chen, Tifei Yuan and Jie Zhang**

Supplemental Information

Menin Deficiency Induces Autism-like Behaviors by Regulating *Foxg1* Transcription and Participates in *Foxg1*-related Encephalopathy

Kai Zhuang, Lige Leng, Xiao Su, Shuzhong Wang, Yuemin Su, Yanbing Chen, Ziqi Yuan, Zi Liu, Jieyin Li, Wenting Xie, Sihan Yan, Yujun Xia, Han Wang, Huifang Li, Zhenyi Chen, Tifei Yuan, Jie Zhang

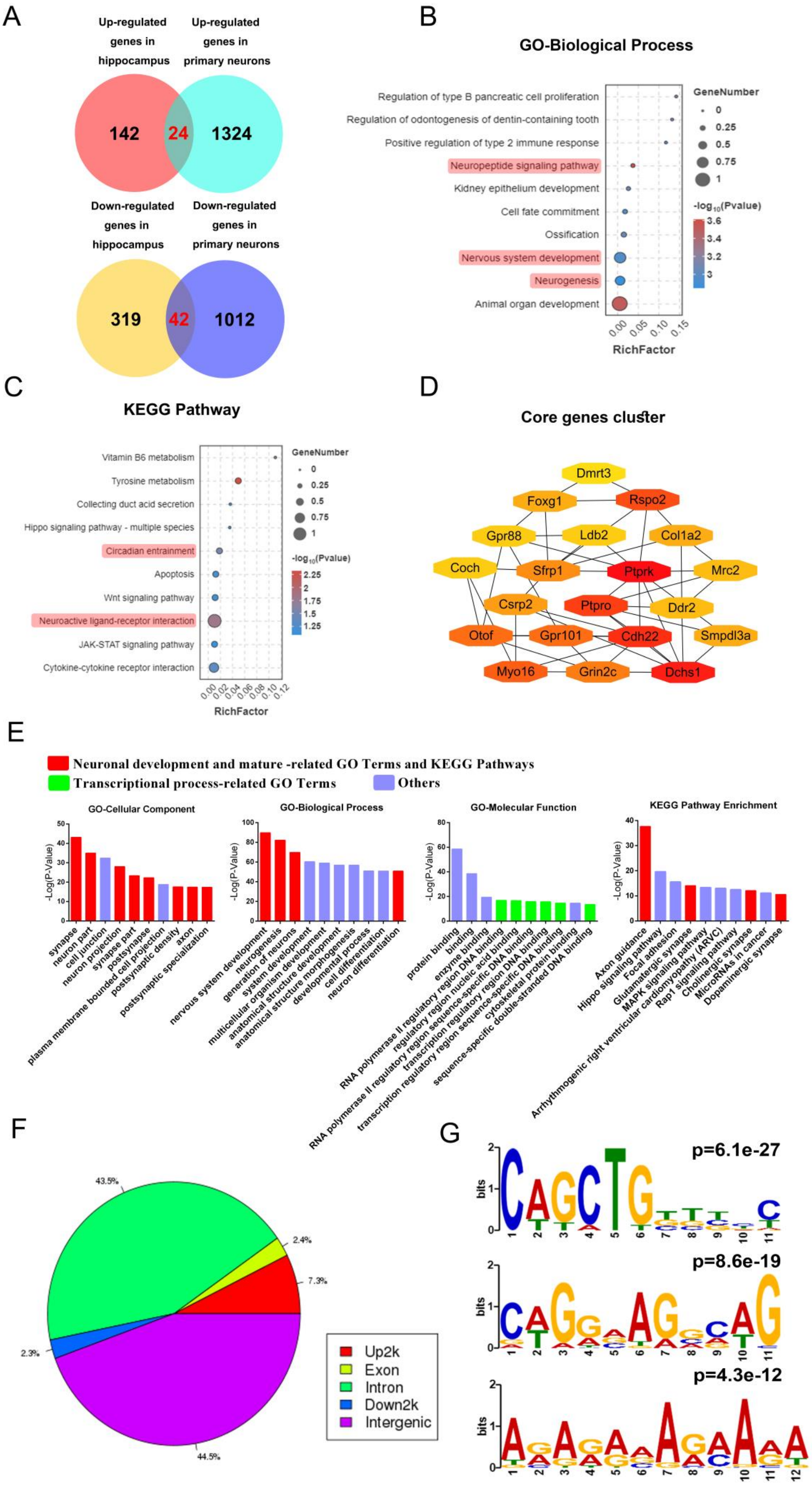
A
Up-regulated genes in hippocampus
Up-regulated genes in primary neurons
142
24
1324
Down-regulated genes in hippocampus
Down-regulated genes in primary neurons
319
42
1012
B
GO-Biological Process
Regulation of type B pancreatic cell proliferation
Regulation of odontogenesis of dentin-containing tooth
Positive regulation of type 2 immune response
Neuropeptide signaling pathway
Kidney epithelium development
Cell fate commitment
Ossification
Nervous system development
Neurogenesis
Animal organ development
GeneNumber
0
0.25
0.5
0.75
1
-log10(Pvalue)
3.6
3.4
3.2
3
0.00
0.05
0.10
0.15
RichFactor
C
KEGG Pathway
Vitamin B6 metabolism
Tyrosine metabolism
Collecting duct acid secretion
Hippo signaling pathway - multiple species
Circadian entrainment
Apoptosis
Wnt signaling pathway
Neuroactive ligand-receptor interaction
JAK-STAT signaling pathway
Cytokine-cytokine receptor interaction
GeneNumber
0
0.25
0.5
0.75
1
-log10(Pvalue)
2.25
2
1.75
1.5
1.25
0.00
0.02
0.04
0.06
0.08
0.10
0.12
RichFactor
D
Core genes cluster
Dmrt3
Foxg1
Rspo2
Gpr88
Ldb2
Col1a2
Coch
Sfrp1
Ptprk
Mrc2
Csrp2
Ptpro
Ddr2
Otof
Gpr101
Cdh22
Smpdl3a
Myo16
Grin2c
Dchs1
E
Neuronal development and mature -related GO Terms and KEGG Pathways
Transcriptional process-related GO Terms
Others
GO-Cellular Component
-Log(P-Value)
synapse
neuron part
cell junction
neuron projection
synapse part
postsynapse
plasma membrane bounded cell projection
postsynaptic density
axon
postsynaptic specialization
GO-Biological Process
nervous system development
neurogenesis
generation of neurons
system development
multicellular organism development
anatomical structure development
anatomical structure morphogenesis
developmental process
cell differentiation
neuron differentiation
GO-Molecular Function
protein binding
binding
enzyme binding
RNA polymerase II regulatory region DNA binding
regulatory region nucleic acid binding
RNA polymerase II regulatory region sequence-specific DNA binding
transcription regulatory region DNA binding
transcription regulatory region sequence-specific DNA binding
cytoskeletal protein binding
sequence-specific double-stranded DNA binding
KEGG Pathway Enrichment
Axon guidance
Hippo signaling pathway
Focal adhesion
Glutamatergic synapse
MAPK signaling pathway
Arrhythmogenic right ventricular cardiomyopathy (ARVC)
Rap1 signaling pathway
Cholinergic synapse
MicroRNAs in cancer
Dopaminergic synapse
F
43.5%
2.4%
7.3%
2.3%
44.5%
Up2k
Exon
Intron
Down2k
Intergenic
G
p=6.1e-27
p=8.6e-19
p=4.3e-12
bits

Figure S1. Multi-omics analysis reveals that menin plays an important tole in the development of the nervous system (Related to Figure 1). A) Venn diagram shows the overlap between differential genes in *Men1*-CcKO mouse hippocampus (vs. Control mice) and in *Men1*-KO primary neurons (vs. WT primary neurons). The above represents the overlap of concordant up-regulation (n=24) and the below represents concordant down-regulation (n=42). **B)** The cluster analysis of KEGG indicates that the common genetic changes are intimately related to nervous system development in RNA-seq from two transcriptional profiles. **C)** GO-biological process indicates that the common genetic changes are intimately related to nervous system development in RNA-seq from two transcriptional profiles. **D)** The core genes (Top 20) of 66 differential genes. From yellow to red, the corresponding gene has a higher weight in network. **E)** The top 10 GO enriched terms (including cellular compound, biological process, molecular function) and top 10 KEGG pathway contributing to menin function were determined by menin-specific binding genomic regions. Red columns represent neuronal development and mature-related GO terms and KEGG pathway, green columns represent transcriptional process-related GO terms and blue columns represent other terms and pathways. **F)** Genomic distribution of menin peaks in DIV10 primary neurons. **G)** The top 3 of menin-associated binding motifs were identified. All motifs were identified by MEME software (http://meme-suite.org/).

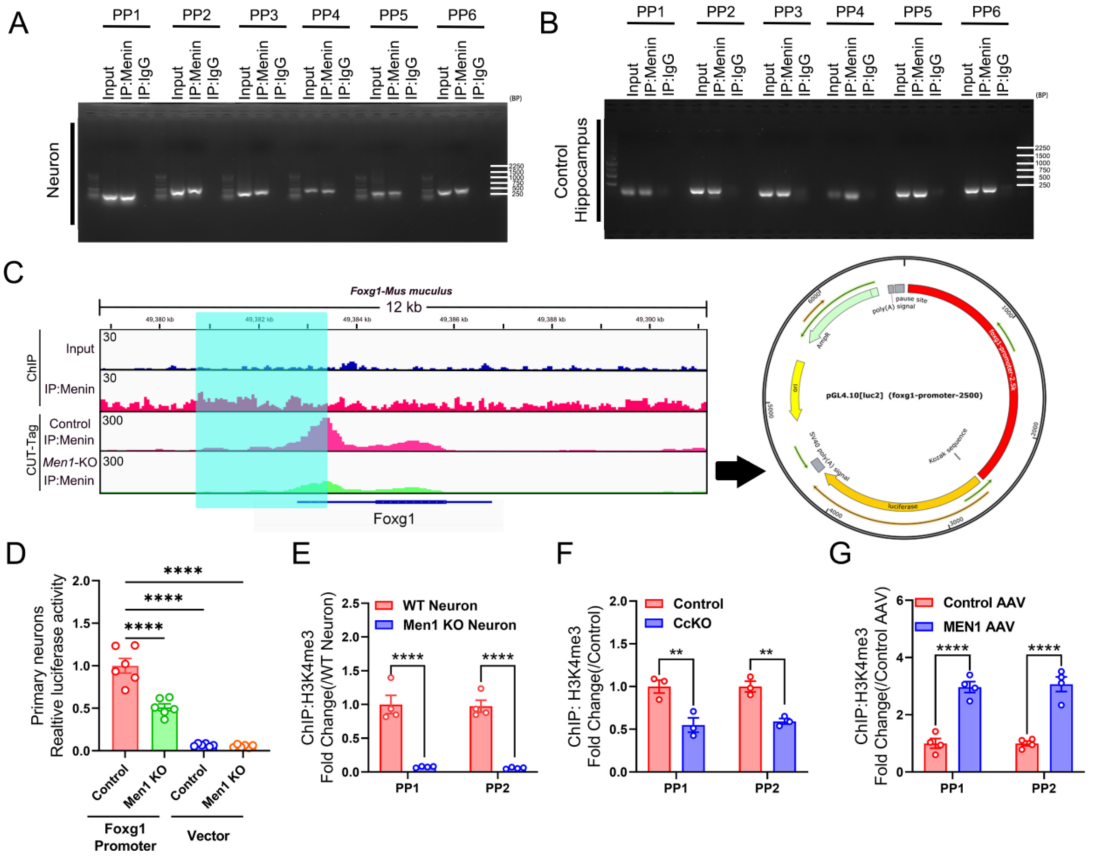

Figure S2. Menin affects the modification of H3K4me3 in *Foxg1* promoter region, resulting in transcription disorder of *Foxg1* (Related to Figure 2). A) The products of the ChIP assay were

verified by DNA gel electrophoresis. The samples from primary neurons of Figure 2B. **B)** The products of the ChIP assay were verified by DNA gel electrophoresis. The samples from the Control mice of Figure 2C were resolved on 1.5 % agarose gels. **C)** According to the region from 2,000 bps upstream of the *Foxg1* transcription start site (TSS) (−2000) to 500 bps in its 5'-untranslated region (UTR) (+500), PGL 4.10-Foxg1 promoter-2500bps was constructed for assessing the luciferase activity. **D)** Transcription activity of *Foxg1* was inhibited in *Men1*-KO primary neurons following transfection with indicated luciferase plasmids (n = 6 samples per group). **E)** H3K4me3-enriched DNA fragments were detected after the ChIP assay in *Men1*-KO and WT primary neurons. (n = 4 experimental replicates per group). **F)** H3K4me3-enriched DNA fragments were detected after the ChIP assay in the hippocampus of 1-month-old *Men1*-CcKO and Control littermates. (n = 4 experimental replicates per group). **G)** H3K4me3-enriched DNA fragments were detected after the ChIP assay in the hippocampus of *MEN1* AAV-treated mice and Control AAV-treated mice. (n = 4 experimental replicates per group).(Data is represented by mean ±SEM.*p < 0.05, **p < 0.01, ***p< 0.001 and ****p< 0.0001 indicate significance between the two indicated groups. ***E, F, G***: two-way ANOVA with Tukey post hoc test. ***D***: one-way ANOVA with Tukey post hoc test.)

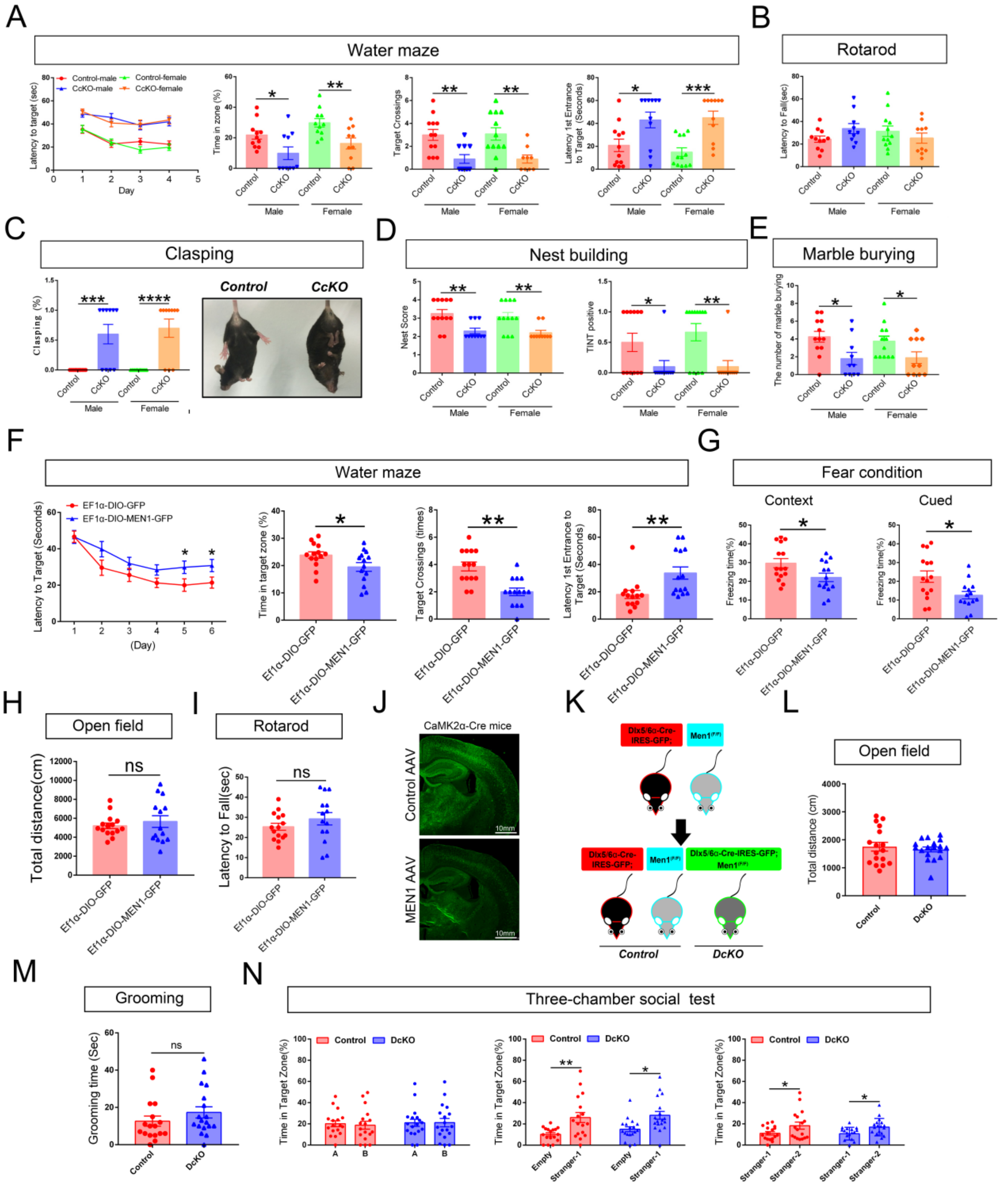

Figure S3. Excitatory neuron-specific deletion and overexpression of *Men1* in mice both resulted in ASD-like behaviors and cognitive defects, while Men1-DcKO showed no changes (Related to Figure 3). A) Morris water maze tests were employed to the assessment of cognitive competence in the four-groups of mice. The images represent the training curve, the time in the target quadrant, the number of target crossing and the latency first time entrance to target from left to right, respectively. **B)** Rotarod tests were used to assess motor ability in the four-groups of mice. **C)** Abnormal hind limb clasping was observed in *Men1*-CcKO mice, and the corresponding statistical results are shown on the left. **D)** Nest building, has been shown to be sensitive to brain lesions, made by Control and *Men1*-CcKO mice during the full extent of one dark phase. Two important parameters represent nest building ability of mice (left) and whether or not they can utilize raw materials for nest building (right). **E)** Marble burying was used to assess compulsive, rigid,

repetitive behaviors in the four-group mice. **F)** Morris water maze tests were employed to the assessment of cognitive competence in the two-group mice. From left to right, they represent the training curve, the time in the target quadrant, the number of target crossing and the latency first time entrance to target respectively. **G)** During fear conditioning tests, both groups of *MEN1* AAV and Control mice were trained and analyzed for freeze response under contextual and cued stimuli. All mice are males. **H)** We tested the free movements of the two groups of mice in an open field. All mice are males. **I)** *MEN1*-overexpressing mice (*MEN1* AAV) and control group mice were evaluated using rotarod tests. All mice are males. **J)** Representative images showing expression of GFP in Control AAV and *MEN1* AAV after 3 months of AAV injections. (Control AAV: n = 3 slices from 3 mice and *MEN1* AAV: n = 3 slices from 3 mice). **K)** An experimental model was established to specifically delete *Men1* in inhibitory neurons (*Men1*-DcKO). **L)** The free movements of the two groups of male mice in an open field. **M)** Grooming tests were used to assess compulsive, rigid, repetitive behaviors in both groups of male mice. **N)** Three-chamber social tests were employed to analyze the social behavior in both groups of male mice. including adaptive exploration stage (left), social novelty tests (middle) and social memory tests (right). All used mice are male. (**A-E**: Control-male group: n = 12; Control-female group: n = 12; *Men1*-CcKO-male group: n = 10; *Men1*-CcKO-female group: n = 10. **F-I**: Control AAV: n = 14; *MEN1* AAV: n = 14. **J-M**: Control: n = 17; *Men1*-DcKO: n = 18. Data is represented by mean ±SEM. ns: not significant. *p < 0.05, **p < 0.01, ***p< 0.001 and ****p< 0.0001 indicate significance between the two indicated groups. ***A, B, C, D, E:*** one-way ANOVA with Tukey post hoc test. ***N:*** two-way ANOVA with Tukey post hoc test. ***F, G, H, I, L, M***: unpaired t-test.)

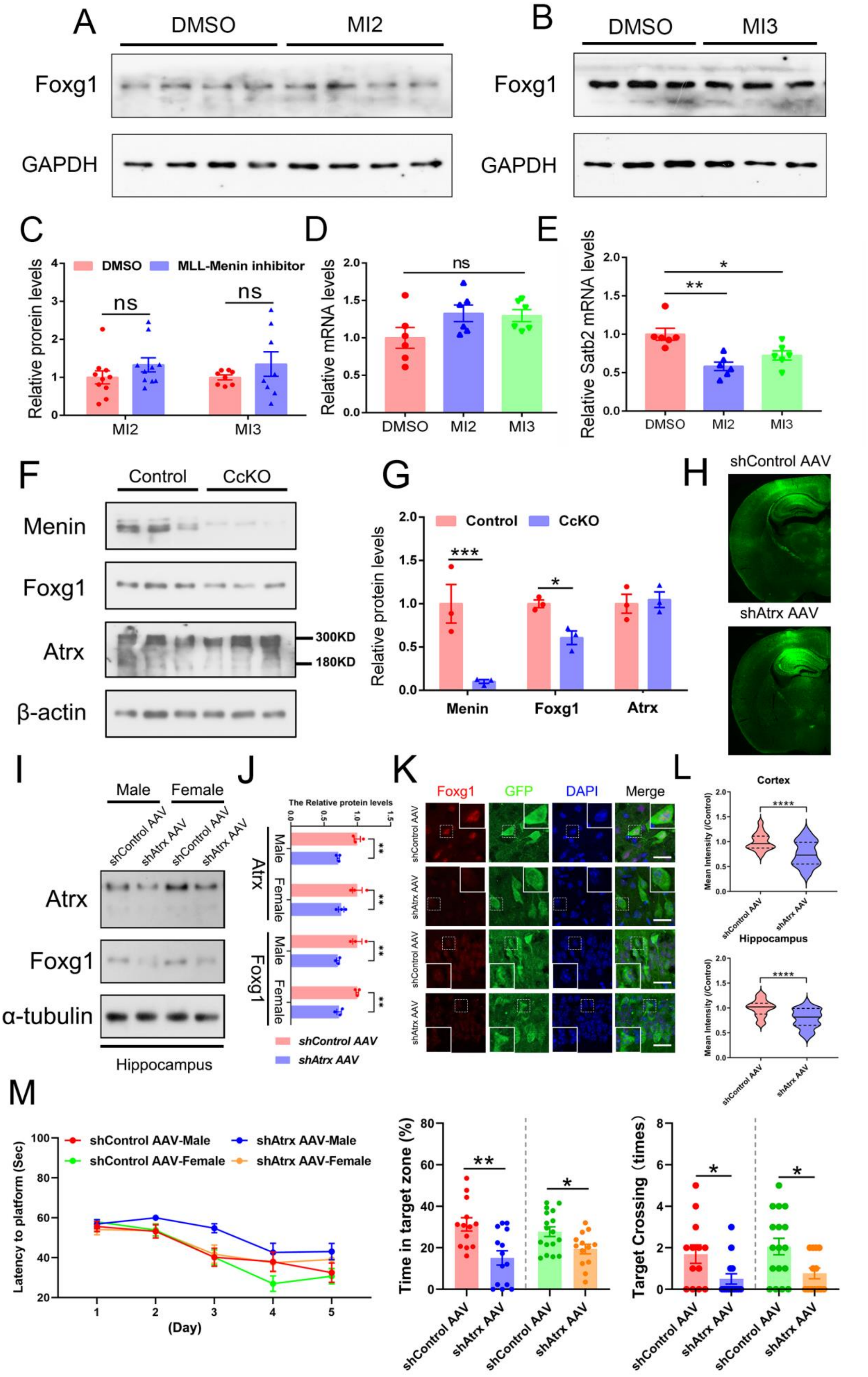
A
DMSO
MI2
Foxg1
GAPDH
B
DMSO
MI3
Foxg1
GAPDH
C
DMSO
MLL-Menin inhibitor
Relative prorein levels
ns
ns
MI2
MI3
D
Relative mRNA levels
ns
DMSO
MI2
MI3
E
Relative Satb2 mRNA levels
*
**
DMSO
MI2
MI3
F
Control
CcKO
Menin
Foxg1
Atrx
300KD
180KD
β-actin
G
Control
CcKO
Relative protein levels
***
*
Menin
Foxg1
Atrx
H
shControl AAV
shAtrx AAV
I
Male
Female
shControl AAV
shAtrx AAV
shControl AAV
shAtrx AAV
Atrx
Foxg1
α-tubulin
Hippocampus
J
The Relative protein levels
Atrx
Male
Female
Foxg1
Male
Female
**
shControl AAV
shAtrx AAV
K
Foxg1
GFP
DAPI
Merge
shControl AAV
shAtrx AAV
shControl AAV
shAtrx AAV
L
Cortex
Mean Intensity (/Control)
****
shControl AAV
shAtrx AAV
Hippocampus
Mean Intensity (/Control)
****
shControl AAV
shAtrx AAV
M
shControl AAV-Male
shAtrx AAV-Male
shControl AAV-Female
shAtrx AAV-Female
Latency to platform (Sec)
(Day)
Time in target zone (%)
**
*
shControl AAV
shAtrx AAV
shControl AAV
shAtrx AAV
Target Crossing (times)
*
*
shControl AAV
shAtrx AAV
shControl AAV
shAtrx AAV

Figure S4. Inhibition of the interaction between MLLs and menin did not affect *Foxg1* expression, while *Atrx*-silencing affected the expression of *Foxg1* in mouse brain and cognition (Related to Figure 4). A) MI2, an inhibitor of menin-MLL, was employed to treat DIV 10 primary neurons. (n = 3 independent experiments and 10 samples per group). **B)** MI3, an inhibitor of menin-MLL, was employed to treat DIV 10 primary neurons. (n = 2 independent experiments and 8 samples per group). **C)** The results corresponding to (A) and (B) were analyzed and quantified. **D)** *Foxg1* mRNA levels were detected in the treatment group of menin-MLL inhibitors and Control group. (n = 6 samples per group). **E)** *Satb2* mRNA levels were detected in the treatment group of menin-MLL inhibitors and in Control group. (n = 3 samples per group). **F)** Menin, Foxg1 and Atrx protein levels were detected in *Men1*-CcKO mouse brain and Control mouse brain. (n = 3 samples per group). **G)** The gray level of three proteins was analyzed and quantified. **H)** Representative images showing expression of GFP in shControl AAV and *shAtrx* AAV after 2 months of AAV injections. (n = 3 slice from 3 mice per group). **I)** Atrx and Foxg1 protein levels were detected in the hippocampus of both male and female mice injected with shControl AAV and *shAtrx* AAV. **J)** The corresponding statistical results for **(I)** are shown. (n = 3 samples per group). **K)** Representative images of Foxg1 immunofluorescence in shControl AAV group and in sh*Atrx* AAV group are provided. The red channel is Foxg1 and the green is GFP. Scale bar, 20 μm. Dashed white boxes indicate representative co-expression cells. Insets marked by yellow arrows show enlarged cells (n = 30 GFP-positive cells per group. shControl AAV group: n=6 slices from 3 mice; *shAtrx* AAV group: n=6 slices from 3 mice. All mice are males.). **L)** The corresponding statistical results for **(K)** are shown, respectively. The above graph is cortical statistic results, the below graph is hippocampal statistic results. **M)** Morris water maze tests were employed to the assessment of cognitive competence in the four-groups mice. From left to right, they represent the training curve, the time in the target quadrant and the number of target crossing respectively (shControl AAV-male group: n = 13; shControl AAV-female group: n = 17; *shAtrx* AAV-male group: n = 14; *shAtrx* AAV-female group: n = 13). (Data is represented by mean ±SEM. ns: not significant. *$p < 0.05$, **$p < 0.01$ and ***$p < 0.001$ indicate significance between the two indicated groups. ***C, G, J, M***: two-way ANOVA with Tukey post hoc test. ***D, E***: one-way ANOVA with Tukey post hoc test. ***L***: unpaired t-test.)

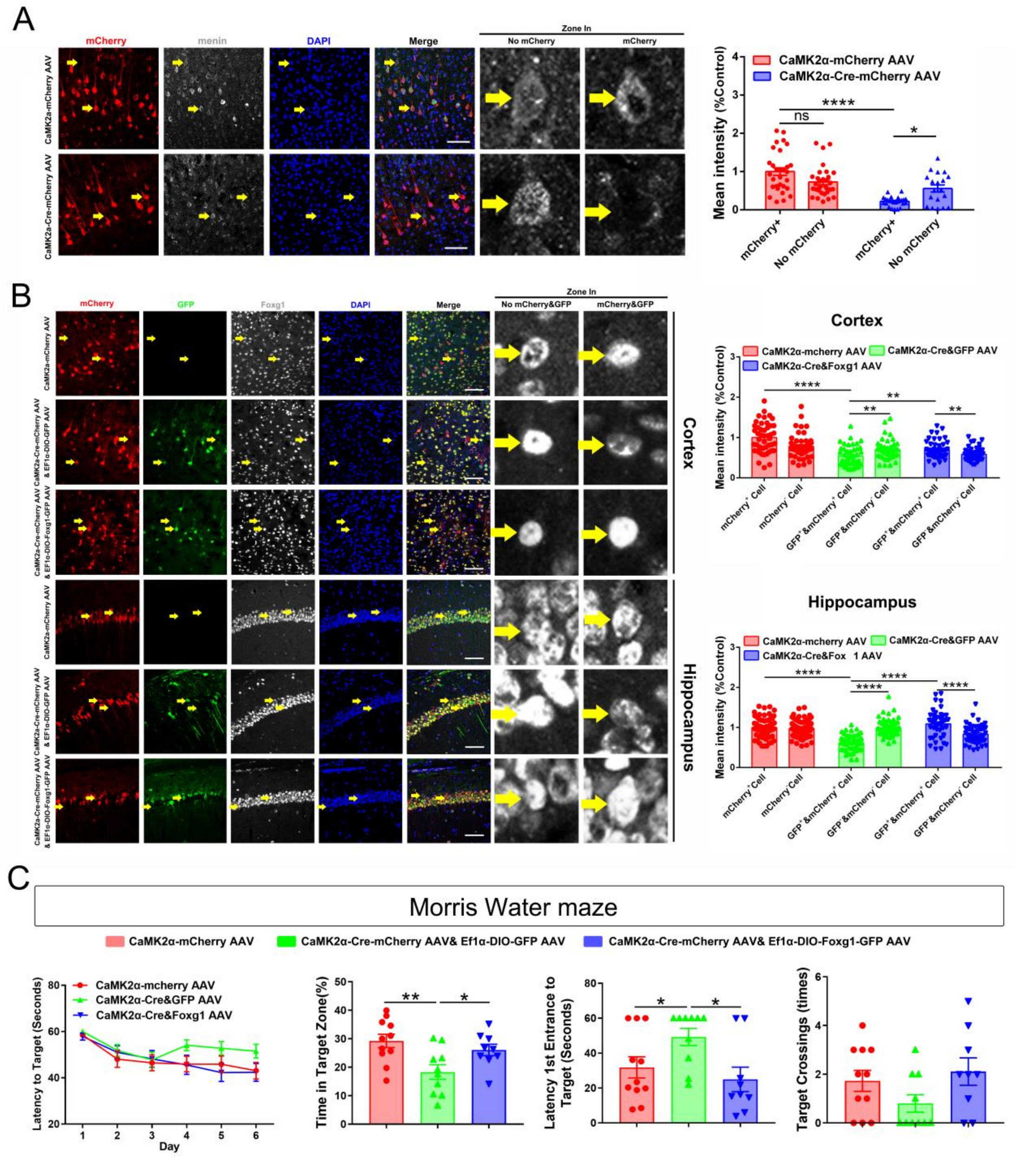

Figure S5. Verification of expression efficiency after combined injection of multiple AAVs (Related to Figure 6). A) The injection of AAV-CaMK2α-Cre-mCherry and AAV-CaMK2α-mCherry was performed in newborn Men1$^{F/F}$ mouse bilateral ventricles. Representative confocal images of brain sections were stained with menin (gray) antibody. The sections were counterstained with DAPI (blue). The red channel is mCherry. Scale bar, 200 μm. mCherry positive cells are representative of cells that express mCherry, while No mCherry indicates cells that do not express mCherry. Insets marked by yellow arrows show enlarged cells. (n = 6 slices from 3 mice per group and the number of mCherry positive cells is 80 per group). **B)** After a combination injection of multi-AAVs, representative confocal images of brain sections were stained with Foxg1 (gray) antibody. The section was counterstained with DAPI (blue). The red channel is mCherry, while the green channel is GFP. Scale bar, 200 μm. mCherry+ indicates mCherry positive cells while mCherry- indicates mCherry negative cells. Similarly, GFP+ indicates GFP positive cells and GFP- indicates GFP negative cells. Insets marked by yellow arrows show enlarged cells. (n =

12 slices from 3 mice per group and the number of mCherry or GFP&mCherry positive cells is 160 per group). **C)** Morris water maze tests, for statistical results represent the training curve, the time in the target quadrant, the number of target crossing and the latency first time entrance to target, respectively (All mice are males. CaMK2α-mCherry AAV: n = 15; CaMK2α-Cre-mCherry AAV& GFP AAV: n = 15; CaMK2α-Cre-mCherry AAV& *Foxg1* AAV: n = 15. Data is represented by mean ±SEM. ns: not significant. *p < 0.05, **p < 0.01 and ****p< 0.0001 indicate significance between the two indicated groups. ***A, B:*** two-way ANOVA with Tukey post hoc test. ***C***: one-way ANOVA with Tukey post hoc test.).
